# Effectiveness of eHealth interventions for improving medication adherence of organ transplant patients: A systematic review and meta-analysis

**DOI:** 10.1371/journal.pone.0241857

**Published:** 2020-11-05

**Authors:** Hyejin Lee, Byung-Cheul Shin, Ji Min Seo

**Affiliations:** 1 College of Nursing, Pusan National University, Yangsan, South Korea; 2 School of Korean Medicine, Pusan National University, Yangsan, South Korea; 3 Pusan National University Korean Medicine Hospital, Yangsan, South Korea; Witten/Herdecke University, GERMANY

## Abstract

**Background:**

Organ transplantation is the most effective treatment for patients with end-stage organ failure. It has been actively carried out all over the world. Recently, eHealth interventions have been applied to organ transplant patients. This systematic review and meta-analysis aimed to evaluate the effects of eHealth interventions for improving medication adherence in organ transplant patients as compared to usual or conventional care alone.

**Methods:**

We searched MEDLINE via PubMed, Excerpta Media dataBASE (EMBASE), the Cochrane Register Controlled Trials, the Cumulative Index to Nursing and Allied Health Literature (CINAHL), PsycINFO, and six domestic Korean databases to identify randomized controlled trials (RCTs) published up to April 17, 2020. Two reviewers independently selected relevant studies and extracted data. The quality and bias of the identified studies were assessed. To estimate the effect size, a meta-analysis of the studies was performed using the Cochrane Collaboration software Review Manager 5.3. PRISMA guidelines were followed. When statistical heterogeneity was greater than 80%, narrative synthesis was performed.

**Results:**

Of the 1,847 articles identified, seven RCTs with a total of 759 participants met the inclusion criteria. The risk of bias assessment showed that the blinding of participants and personnel was high. In six studies, medication adherence (effect size = -0.18–1.30) and knowledge scores were not significantly different between those receiving eHealth interventions and the controls.

**Conclusions:**

Our findings suggest that eHealth interventions were similar to standard care or advanced care for improving medication adherence, and they faired equally well for improving medication knowledge. Therefore, eHealth interventions can be used for medication adherence of organ transplant patients. More research is needed to provide well-designed eHealth intervention to improve the medication adherence and knowledge of organ transplant patients.

**Protocol registration number:**

CRD42017067145 16/05/2017

## Introduction

Organ transplantation is the most effective treatment for patients with end-stage organ failure [1]. In recent years, organ transplantation has been actively carried out all over the world. In 2018, 35,547 transplants were performed in the US [2], and 3,908 transplants were performed in South Korea [3]. While the procedures are somewhat common, they still involve several risks and potential complications for patients. Graft/transplant rejection is the primary complication, and patients should take immunosuppressants to prevent this [4]. Immunosuppressants are important to inhibit rejection and keep the transplanted organs functioning normally [4]. Up to 60% of late acute rejection and about 30–35% of graft loss is due to medication non-adherence [5–8].

The rate of non-adherence to immunosuppressant regimens has been shown to be high. For example, it was reported that 23.1−42.6% of kidney transplant patients [9, 10] and 30.0% of patients five years post-lung transplantation [11] did not adhere to immunosuppressant medication instructions. In addition, a meta-analysis conducted on all recipients of solid organ transplants from 1981 to 2005 found that the average rate of immunosuppressant non-adherence was 22.6% [12].

Recipients of organ transplants are required to take lifelong immunosuppressants after discharge, so they must have adequate knowledge of these medications. The higher the knowledge level, the better the patient’s adherence to medication and treatment instructions, and the better the outcome of treatment [13]. Previously, discharge education was mainly provided to improve the knowledge of organ transplant patients [14].

Recently, eHealth interventions have been implemented to improve medication adherence or knowledge by organ transplant and other patients [15–23]. Further, previous research has reported that patients with organ transplants are an ideal population for utilizing eHealth tools, such as mobile apps [15]. eHealth interventions employ digital processes and communication methods to be delivered through electronic devices such as a mobile phone or personal computer (PC) [16, 17]. The information provided can offer health education; monitor, record, and transmit data about health behaviors and indicators, such as blood pressure or blood sugar levels; and give reminders, feedback, and counseling to patients through methods such as mobile apps, the Internet, and electronic devices [16, 17]. Studies applying various eHealth interventions for improving medication adherence by organ transplant patients have been published, including research on developing mobile apps for kidney transplant patients [18] and methods used to monitor mobile phones, computers, or reminder systems in connection with transplant patients [19–23].

As more information and communication technologies are expected to be applied in the medical field, it is necessary to comprehensively analyze the effects of eHealth interventions for medication adherence that directly affect the survival rate of organ transplant patients. Although the details of the use of eHealth interventions for improving medication adherence by organ transplant patients are currently of great interest, there have been no systematic review and meta-analysis studies published focusing on the effects of eHealth interventions. Additionally, meta-analyses of the various interventions applied with organ transplant patients have been conducted, and the results have been effectively reported [24–26], but no study to date has separately analyzed eHealth interventions. Therefore, this study aimed to evaluate the effects of eHealth interventions on improving medication adherence and knowledge when applied to organ transplant patients, as compared to typical or conventional care, and to conduct a systematic review and meta-analysis to provide guidance on how eHealth interventions for organ transplant recipients should be organized to be effective.

## Methods

### Protocol and registration

The study was approved by PROSPERO (S1 Appendix), protocol registration number: CRD42017067145 16/05/2017. The review was conducted in accordance with PRISMA guidelines [27] (S2 Appendix). Ethical approval for review studies was not required by the authors’ respective institutions.

### Eligibility criteria

This systematic review included randomized controlled trials (RCTs) that evaluated the effects of providing eHealth interventions for improving organ transplant recipients’ medication adherence. Studies of adult patients (18 years or older) who had been discharged following organ transplantation were eligible for inclusion. Participants under 18 years were excluded, as they were determined to be unable to independently adhere to medication regimens, such that their guardians or parents might be primarily responsible for their medication adherence. This review included patients regardless of gender and race. The number of transplant organs was not limited and only those involving solid organ transplants were selected.

For our study interventions, we included eHealth interventions. Prior studies using various information and communication technology devices were considered. For our study controls, we included research control interventions that employed any reasonable interventions or usual care and did not involve the provision of eHealth interventions for improving medication adherence.

As for outcomes, we only included medication adherence and medication knowledge outcome measurements from studies involving eHealth interventions for transplant recipients as objective measures (via clinical measures such as Tacrolimus serum concentration levels, pill counts, and prescription refill data) and subjective measures (e.g., self-report questionnaires, such as the Health Habits Survey (HHS) [22] and Basel Assessment of Adherence to Immunosuppressive Medication Scale [28, 29]). We excluded non-original studies, abstract-only published studies, and reviews.

### Information sources and searches

Five worldwide electronic databases (MEDLINE via PubMed, EMBASE, CINAHL, PsycInfo and Cochrane Library) and six Korean electronic databases (KoreaMed, Korean Medical Database [KMBASE], Korean Studies Information Service System [KISS], National Science Digital Library [NSDL], Korea Institute of Science and Technology Information [KiSTi] and Research Information Service System [RISS]) were searched for studies published up to April 17, 2020. The search terms used for electronic databases were chosen following the PICO format (P: patients, transplantation; I: intervention, eHealth; C: control [any control interventions], O: outcome [medication adherence and medication knowledge outcomes]) and modified as necessary to include equivalent terms for each database. The MEDLINE search terms used are presented in S3 Appendix. Additionally, we manually searched the references listed in the present review article to find further. Language restrictions were not applied [30].

### Study selection

Two independent reviewers (HJL and JMS) screened the titles and abstracts for potentially eligible studies identified by the primary search, and then reviewed the full texts to evaluate their final eligibility. The two authors cross-checked each other’s articles, and, in the case of any disagreement regarding extracted data, a third expert (BCS) was brought into the discussion. Decisions were made based on consensus.

### Data collection process and data items

After selecting articles for inclusion, we extracted the following data along with the intervention characteristics: authors, publication year, publication country, types of organs transplanted, sample size, average patient age, study blinding, intervention, intervention duration, control group intervention, measurement points, outcomes, and measures as predefined.

### Risk of bias

Quality assessment was conducted using the Cochrane risk of bias (ROB) criteria tools [31]. We ranked each item as belonging to one of ROB three levels—“Low,” “Unclear,” or “High”—following the Cochrane guidelines for ROB assessment in seven domains: random sequence generation, allocation concealment, blinding of participants, blinding of outcome assessment, incomplete outcome data, selective reporting, and intention to treat [31]. To gauge the participant blinding in the included studies, we categorized a study as having a low ROB when the blinding of patients was clearly identified. To assess the ROB on outcome assessors, we concluded that a study had a low ROB if the authors plainly reported that they blinded the outcome assessors, or the outcome measure was assessed by blinded assessors only. Studies were rated as having an unclear ROB if the outcome measures were built from both subjective and objective assessments, and we could not clearly judge whether the outcome assessor was blinded or not. Regarding the reporting of incomplete outcome data, a study was rated as having a low ROB if it satisfied three criteria: (1) the number of attrition cases and the causes were clearly reported in each group, (2) the attrition rates were similar between groups, and (3) the percentage of withdrawals and drop-outs did not exceed 20% in the short-term and 30% in the long-term follow-up periods [32]. If there were no dropouts in studies, they were rated as having a low ROB. The other bias that was assessed was an analysis of intention to treat. When we confronted problems referring to the assessment of ROB, we solved them by having a consensus-based discussion among reviewers.

### Summary measures and synthesis of results

All outcome measurements were extracted as means and standard deviations (or transformed) or number of events and total sample size. Outcome measures from at least three months after the start of the intervention were used in data pooling.

The risk estimates (relative risk: RR) with 95% confidence intervals (CIs) were calculated for dichotomous data and standardized mean differences (SMDs) with 95% CIs were employed for continuous data because different scales had been used among studies. For studies with more than one control group, we restricted our analyses to comparing an eHealth intervention group and non-eHealth control groups. The statistical heterogeneity was assessed using the *I*^2^ test by Higgins [33] or Cochrane Q statistics. We determined that heterogeneity existed if the *I*^2^ was above 50% [33] or the Cochrane Q statistics presented as *P* < .10. However, the cut-off point of *I*^2^ to assess heterogeneity was presented differently depending on the research. Some researchers have argued that *I*^2^ values should be around the 25% mark [34]. Our review used the random effect model to deal with heterogeneity that employs variation factors as correction weight. A random effect model can assess both within- and between-study variability and consider the clinical and statistical heterogeneity. Narrative synthesis was performed when the statistical heterogeneity was too high for 80% or more, or when it was not possible to use meta-analysis for a single study. These results were described as forest plots without a pooled estimate. Meta-analysis and narrative synthesis were both performed using the Review Manager software (version 5.3 for Mac; the Nordic Cochrane Centre, Copenhagen, Denmark).

## Results

### Study selection

**Fig 1:**
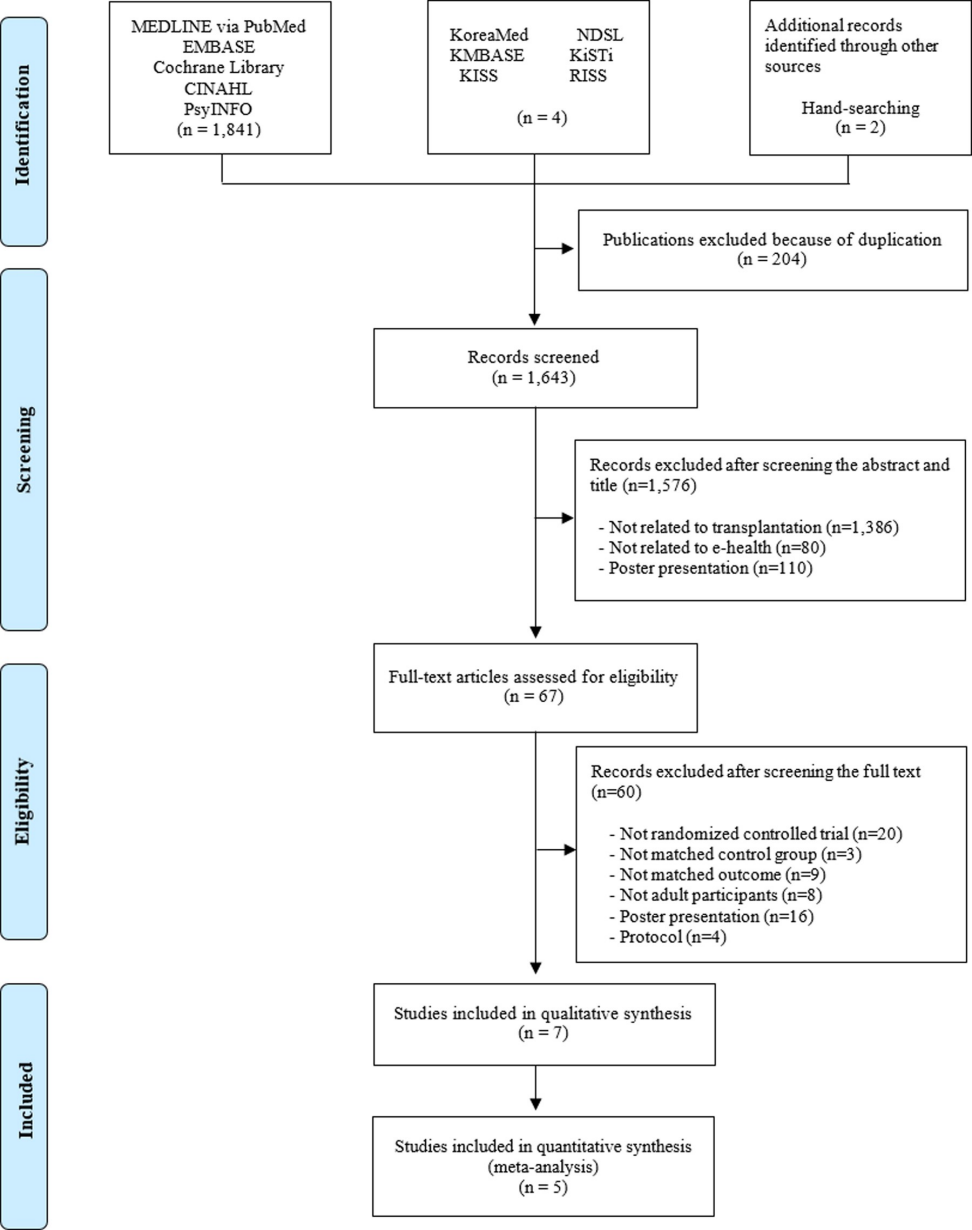
Flowchart of the RCT selection process.

Our search terms yielded 1,847 records, including 1,748 from MEDLINE via PubMed, EMBASE, Cochrane Library, CINAHL and PsycINFO, and six from domestic Korean databases and relevant journals. After removing duplicated studies, 1,643 records were screened. Based on titles and abstracts, 1,576 records were excluded for not meeting the inclusion criteria. We retrieved and reviewed 67 full articles. After full-text reviews, 60 records were excluded because 20 of the articles were not RCTs, and 40 did not meet the inclusion criteria due to several reasons that have been summarized in Fig 1 as recommended by PRISMA guidelines [35]. Finally, a total of seven RCTs [19, 21, 22, 28, 29, 36, 37] were included in our review. The included studies are listed in S4 Appendix.

### Study characteristics

The characteristics of the seven studies [19, 21, 22, 28, 29, 36, 37] selected in this review were analyzed separately and are summarized in Table 1.

**Table 1:** Descriptive summary of included studies.

Study First author [ref.] (year, country)	Organ	Sample size (n)		Age (mean / median)		Blinding	Intervention (duration)	Control	Measurement point (months)	Outcomes (measures)
		I	C	I	C					
DeVito Dabbs et al. [22] (2016, USA)	Lung	99	102	62.0	62.0	Single-blind (data collectors)	**Mobile application for self-management** (12 months)	Scripted discharge instructions	2, 6, 12	1) Medication adherence—Subjective: self-report questionnaire (HHS)
							Intervention group received discharge instructions and a smartphone with custom programs to self-record daily health indicators (vital signs, symptoms), view graphical displays of trends, and receive automatic feedback messages if health indicators were critical.			
Han et al. [29] (2019, Korea)	Kidney	70	66	45	43	Single-blind (survey assessors)	**Mobile application for medication management** (6 months)	Education on the importance of adherence	1, 3, 6	1) Medication adherence—Subjective: self-report questionnaire (BAASIS, VAS)—Objective: electronic monitoring (medication bottle with MEMS V prescription container lids)
							Intervention group was provided with mobile application for medication management. The features of the application included visual and auditory reminders that reported the state of the medication, monitored the state of the participant’s medication, and provided education on immunosuppressants.			
Harrison et al. [36] (2017, Canada)	Solid organ (multiple)	104	105	48.1	49.6	Non-blinded	**Web-based education** (3 months)	Pharmacist-led program	3	1) Medication adherence—Subjective and objective: self-report questionnaire and immunosuppressant drug level of blood (MACS) 2) Medication knowledge (self-report questionnaire)
							Educational content aligns with the self-management program and primarily focused on patient understanding of medications.			
McGillicuddy et al. [37] (2013, USA	Kidney	9	10	42.4	57.6	Not reported	**Mobile phone-based medication monitoring** (3 months)	Education related to post- transplantation medical care	1, 2, 3	1) Medication adherence—Objective: Russell et al.’s adherence score
							Intervention group received customizable reminder signals (light, chime), phone calls, or text messages at the prescribed dosing day and time. They were contacted by text, email, or phone when alerts indicated medication non-adherence. A weekly summary report was delivered via email and summarized each participant’s adherence to medication dosing by a physician.			
Reese et al. [28] (2017, USA)	Kidney	39*	38	50.0	49.0	Double-blind (investigator, statistical analysts)	**Automated medication reminders with wireless pill bottle and physician notification** (6 months)	Wireless pill bottle that provided no alerts	6	1) Medication adherence—Subjective: self-report questionnaire (BAASIS)—Objective: wireless pill bottle openings, blood concentrations of Tacrolimus
							For each participant receiving reminders, a light on the bottle would illuminate, and the cap would chime when the medication should be taken. If adherence decreased to < 90% every 2 weeks, the study coordinator would contact the participant by telephone.			
Sengpiel et al. [19] (2010, Germany)	Lung	28	28	49.5	48.5	Not reported	**Mobile phone-based spirometry monitoring** (6 months)	Home spirometry without Bluetooth	6	1) Medication adherence—Objective: trough levels of immunosuppressive drugs in target range
							Intervention group received a Bluetooth-capable AM1+ home spirometer connected to the patients’ cell phone and a central database server. FEV_1_ digitally displayed to the patient with a traffic-light system (green = 90−100% of FEV_1_ baseline, yellow = 50−90%, red < 50%).			
Suhling et al. [21] (2014, USA)	Lung	30	31	52.0	45.0	Non-blinded	**Tablet/PC-based education** (6 months)	Counseling by a trained nurse using written material on patient medication	6	1) Medication adherence—Subjective: self-report questionnaire (BAASIS, ITBS, Morisky score)—Objective: blood levels of immunosuppression in target range 2) Medication knowledge (self-report questionnaire)
							Education consisted of 30 slides and four video clips totaling 12.75 min about medication.			

*A customized reminder and notification group.

Abbreviations: BAASIS = Basel Assessment of Adherence to Immunosuppressive Medication Scale, C = control, FEV_1_ = Forced Expiratory Volume in 1 second, HHS = Health Habits Survey, I = intervention, ITBS = Immunosuppressant Therapy Adherence Barrier Instrument, MACS = Multidimensional Adherence Classification System, MEMS = Medication Event Monitoring System, VAS = Visual Analog Scale

#### Intervention characteristics

Table 1 shows the characteristics of the interventions used in the seven studies included in this analysis. These interventions utilized mobile application-based self-management [22], mobile applications for medication management [29], web-based education [36], mobile phone-based medication monitoring [37], automated medication reminders with a wireless pill bottle and physician notifications [28], mobile phone-based spirometry monitoring [19], and tablet/PC-based education [21]. Details of the intervention for each study are as follows. In DeVito Dabbs et al.’s study [22], the intervention group received discharge instructions and a smartphone with custom programs to self-record daily health indicators, view graphical displays of trends, and receive automatic feedback messages if health indicators were critical. In Han et al.’s study [29], the intervention group was provided with a mobile application for medication management. The features of the application included reminders that reported on the state of the medication, monitored the state of the participant’s medication, and provided education on immunosuppressants. In Harrison et al.’s study [36], education about medication via a website was provided. In McGillicuddy et al.’s study [37], the intervention group received customizable reminder signals (light, chime), phone calls or text messages at the prescribed dosing day and time. They were also contacted by text, email, or phone when alerts indicated medication non-adherence. In Reese et al.’s study [28], the intervention group received reminders, in which a light on the bottle would illuminate and the cap would chime when the medication should be taken. If adherence decreased to < 90% every 2 weeks, the study coordinator would contact the participant by telephone. In Sengpiel et al.’s study [19], the intervention group received a Bluetooth-capable AM1+ home spirometer connected to the patients’ cell phone and a central database server. Depending on the degree of FEV_1_, the traffic-light system's color would change. In Suhling et al.’s study [21], education about medication was provided with tablets/PCs.

#### Outcomes

Outcome variables were medication adherence (n = 7) [19, 21, 22, 28, 29, 36, 37] and medication knowledge (n = 2) [21, 36]. The seven studies included in this analysis showed that the medication adherence measurement methods were significantly heterogeneous. The definitions of medication adherence described in each study are as follows. DeVito Dabbs et al.’s study [22] measured medication adherence using the HHS, a subjective self-report questionnaire. The HHS comprises taking medications, attending clinical appointments, and completing lab work. Ordinal response formatting was used to indicate how often each element was performed; the responses were then dichotomized to indicate whether the lung transplant recipients met the minimal level of adherence for each element deemed acceptable by the transplant team (e.g., the recipient missed taking their immunosuppressant less than once per month). To arrive at a composite measure of overall adherence, the authors summed the number of elements (from a total of nine). Adherence was dichotomized into high adherers (median of eight and higher) and lower adherers (less than eight). Because the outcomes of medication adherence could not be separated from the full HHS survey results, the full results of the HHS were used in this review.

Han et al.’s study [29] measured outcomes using both objective methods, such as medication bottles with MEMS V prescription container lids (dichotomous data), and subjective methods using BAASIS and VAS self-rated adherence (dichotomous data). The objective study outcome was a binary indicator of cumulative six-month adherence based on electronic monitoring data. Medication adherence was defined as taking medication as prescribed 98% of the time. The BAASIS includes four items that assess the medication us and timing, drug holidays, and dose reduction on a 6-point scale, ranging from “never” (0) to “every day” (5). The VAS score ranged from “never took the medication as prescribed” (0) to “always took the medication as prescribed” (100). Nonadherence was defined as a positive answer to any of the four items (score ≥ 1) using the BAASIS and as a score other than 100 using the VAS. Accordingly, the number of nonadherent patients was calculated on days 28, 90, and 180. For this meta-analysis, values measured with the BAASIS were used.

Harrison et al.’s study [36] measured medication adherence in MACS, a combination of both objective and subjective measurements. The subjective measurement results came from participants reporting when the missed a dose or took their medication late over the previous week. The proportions of missed or late doses were calculated, where “late” was defined as more than 1 hour past the patient’s usual routine. The objective measurement results relied on Tacrolimus blood concentration (dichotomous data). Immunosuppressant levels were collected as per routine practice, and standard deviations of levels within a prescribed dose were calculated. These data were used to classify patients into one of four adherence groups. In this meta-analysis, subjective and objective measurement results were used separately.

McGillicuddy et al.’s study [37] used Russell et al.’s adherence score to objectively measure medication adherence related to medication time (continuous data). The study considered medication adherence to be when participants took their immunosuppressants within three hours of the prescribed dosing time. A dose taken within the three-hour window resulted in a full score for that dosing time, a dose taken outside the three-hour window but within a six-hour window resulted in a half score, and a missed dose resulted in a score of 0. Each participant was assigned a score ranging from 0.0 to 1.0 for each day, and participants’ scores were averaged over each month.

In Reese et al.’s study [28], medication adherence was measured using both objective methods such as pill bottles (dichotomous data) and Tacrolimus blood concentration (continuous data) and subjective methods using BAASIS self-rated adherence (dichotomous data). Medication adherence was defined as the percentage of days with bottle openings as expected from the intended timing of daily Tacrolimus dosing, Tacrolimus blood concentrations, and BAASIS scores, using a validated 5-item self-report questionnaire specific to immunosuppression. In Sengpiel et al.’s study [19], medication adherence was the number of through levels of immunosuppressants in a target range (dichotomous data). In Suhling et al.’s study [21], medication adherence was measured using both objective methods, such as percentage of immunosuppression levels in a target range six months after education (dichotomous data), and subjective methods using the BAASIS, ITBS, and Morisky score (continuous data). The Morisky score with standard deviations was used in the meta-analysis, and consists of four questions related to medication adherence, each of which can be measured from 0−4 points.

Medication knowledge was measured using a questionnaire consisting of the name, dose, and number of immunosuppressants. Of the two studies that adopted medication knowledge as an outcome, one reported continuous data as an indication of a change in the patients’ knowledge score [36], and one used dichotomous data as an indication of the proportion of participants with improved knowledge [21].

### Risk of bias

Of the seven studies, six [19, 21, 22, 28, 29, 36] employed appropriate methods of sequence generation. For example, they employed a random number generator or a computer randomized number generator. Group assignment was adequately concealed in three trials [22, 29, 36], using sealed opaque envelopes or central allocation. Of the seven studies, only three RCTs [22, 28, 29] reported a proper description of assessor blinding and had independent assessors to evaluate outcome measurements. Additionally, two studies [21, 36] reported difficulties in blinding due to realistic problems. None of the study designs included double-blinding of the participants and practitioners.

Regarding incomplete outcome data, we evaluated six studies [19, 21, 22, 28, 36, 37] as having a low ROB. All of them had no missing data or few missing data, and a balanced number of participants in each group (eHealth intervention and control groups). In studies that had missing outcome data, the frequency of and causes for drop-outs in each group were similar. Moreover, the short-term drop-out percentage did not surpass 20% and the long-term rate did not surpass 30%. However, when the intervention was provided for six months in one study [29], attrition occurred in 26.8% of the experimental group and 19.4% of the control group. By comparing patients who stopped participating before 28 days after the intervention began to those who continued to participate after 28 days, it was found that the high drop-out rate reduced the intervention’s overall effect size.

**Fig 2:**
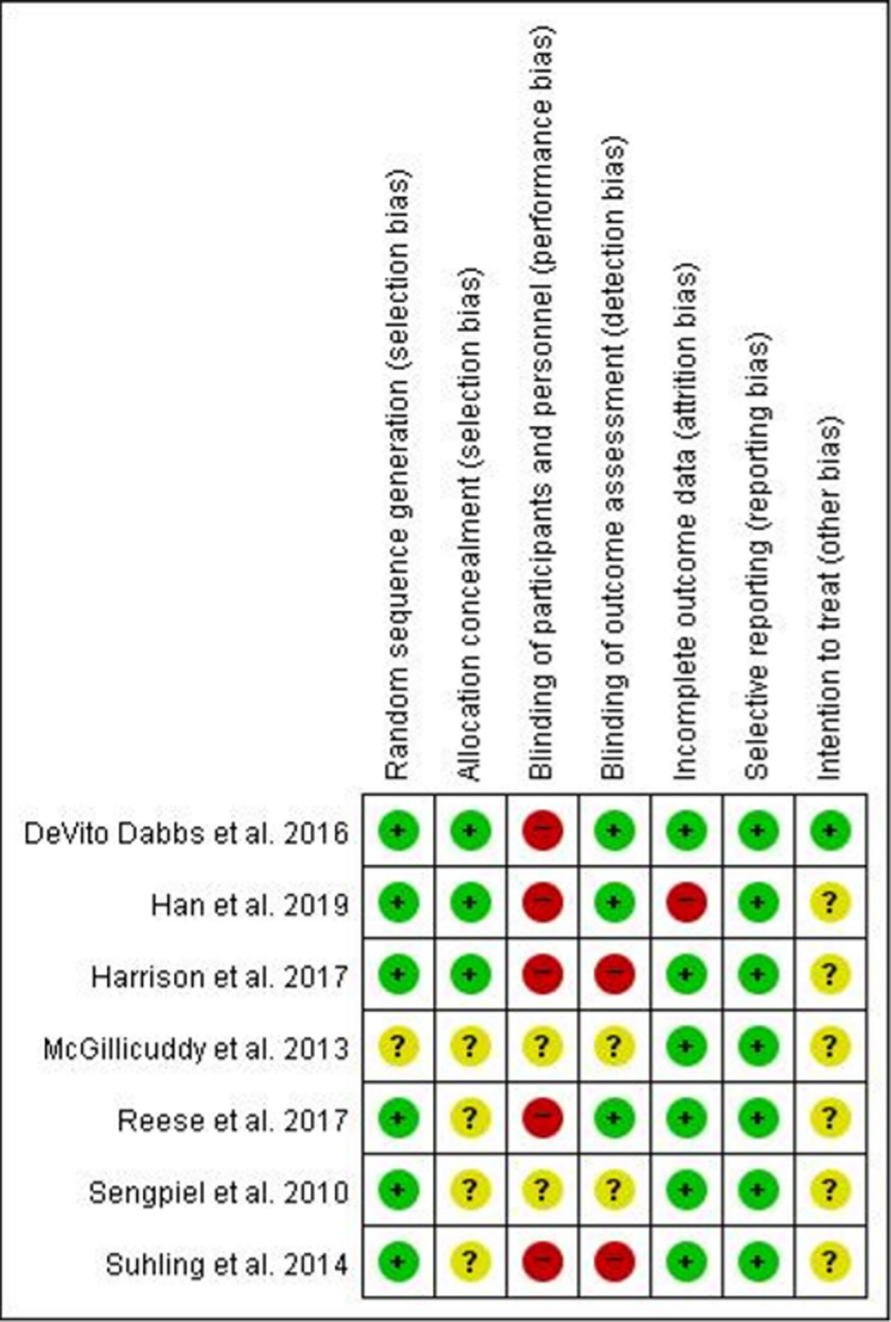
Risk of bias summary.

For the selective outcome reporting, it was impossible to locate and study the protocols of any of the selected studies. Regarding the intention to treat analysis, we evaluated one study [22] which explicitly stated an intention to treat analysis was conducted. In response, we discerned the ROB using the methods reported in each study (Fig 2).

### Results of individual studies and synthesis of results

All seven of the studies measured medication adherence after transplant patients received eHealth interventions. In total, 759 participants were included in the analysis. The measured values were taken at least three months after the start of the intervention. The studies by Harrison et al. [36] and McGillicuddy et al. [37] used values measured at three months, while the other studies [19, 21, 22, 28, 29] used values measured at six months.

Medication adherence measurement methods were identified as being either objective or subjective. Meta-analysis was performed for studies that objectively measured medication adherence and presented dichotomous data. Narrative synthesis was performed for studies that objectively measured medication adherence and presented continuous data or presented results for subjective measurements of medication adherence.

**Fig 3:**
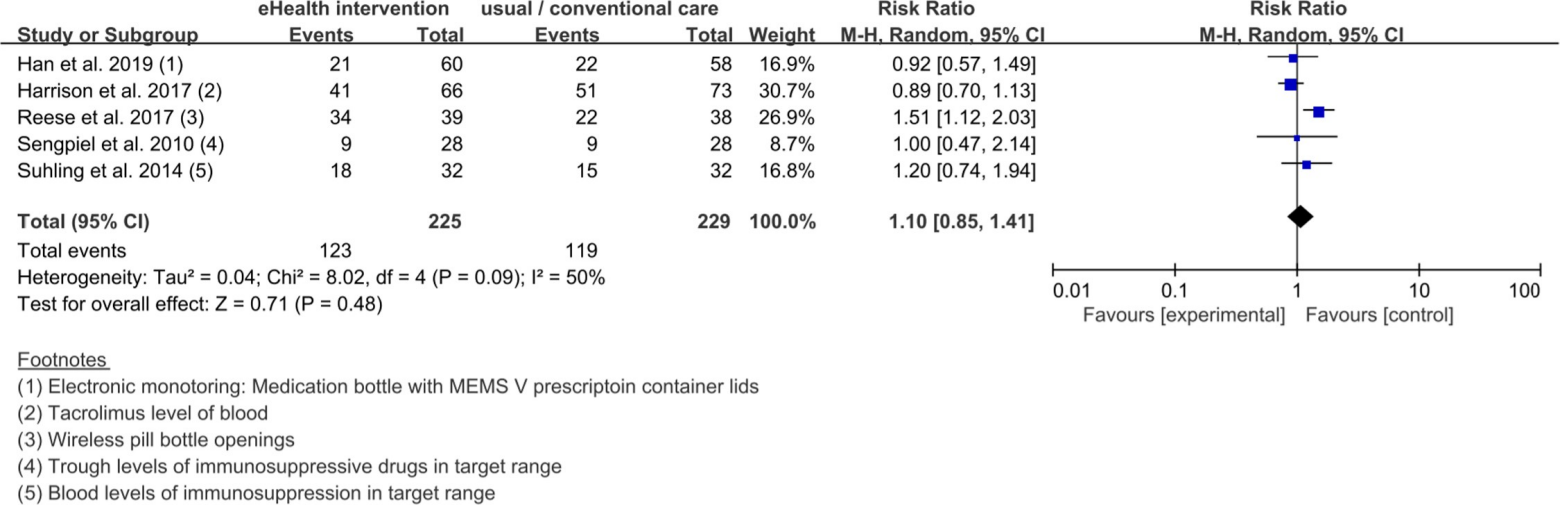
Forest plot of the pooled effect size of eHealth interventions for medication adherence in organ transplant patients. The five studies presented here used objective methods to measure medication adherence in organ transplant patients and presented the results as dichotomous data.

**Fig 4:**
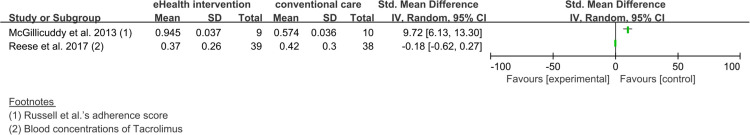
Forest plot without a pooled estimate demonstrating the effectiveness of eHealth interventions for medication adherence in organ transplant patients. The two studies presented here used objective methods to measure medication adherence in organ transplant patients and presented the results as continuous data.

**Fig 5:**
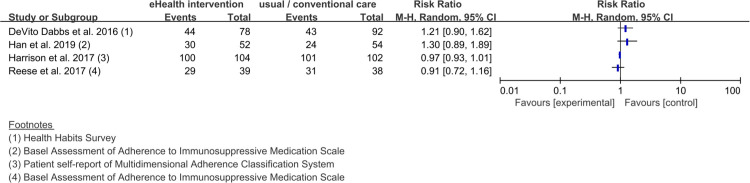
Forest plot without a pooled estimate demonstrating the effectiveness of eHealth interventions for medication adherence in organ transplant patients. The four studies presented here used subjective methods to measure medication adherence in organ transplant patients and presented the results as dichotomous data.

**Fig 6:**
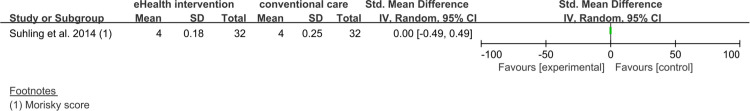
Forest plot without a pooled estimate demonstrating the effectiveness of an eHealth intervention for medication adherence in organ transplant patients. The one study presented here used subjective methods to measure medication adherence in organ transplant patients and presented the results as continuous data.

The studies [19, 21, 28, 29, 36] which objectively measured medication adherence, confirmed that the statistical heterogeneity of the five trials that presented dichotomous data was 50%, and a meta-analysis was conducted (Fig 3). The statistical heterogeneity of the two studies [28, 37] that presented continuous data was measured at 97% and narrative synthesis was performed (Fig 4). For the studies [21, 22, 28, 29, 36] which subjectively measured medication adherence, a narrative synthesis was performed, due to its small number of trials (dichotomous data: four trials (Fig 5) [22, 28, 29, 36], continuous data: one trial (Fig 6) [21]) and high statistical heterogeneity (dichotomous data: 81%).

### Effects of eHealth interventions

#### Medication adherence

The results of the meta-analysis of the five studies using objective measurements and dichotomous data [19, 21, 28, 29, 36] showed that the effects of the eHealth interventions were similar to those of the care provided to the control group at 1.10 (95% CI 0.85 to 1.41) and Z = 0.71 (*P* = .48). The heterogeneity of the overall effect size on medication adherence for eHealth interventions was high (χ^2^ = 8.02, *P* = .09, *I*^2^ = 50%; Fig 3).

Regarding the results of the narrative synthesis of studies with objective measurements and continuous data, in McGillicuddy et al.’s study [37], the effect size of the experimental group provided with mobile phone-based medication monitoring was 9.72 (95% CI 6.13 to 13.30), compared to the control group provided with education. In Reese et al.’s study [28], the effect size of the experimental group provided with automated medication reminders was -0.18 (95% CI -0.62 to 0.27), compared to the control group provided with a wireless pill bottle without an alarm.

Regarding the results of the narrative synthesis of subjective measurements, in DeVito Dabbs et al.’s study [22], the effect size of the experimental group provided with a mobile app intervention was 1.21 (95% CI 0.90 to 1.62), compared to the control group provided with scripted discharge instructions. In Han et al.’s study [29], the effect size of the experimental group provided with a mobile app intervention was 1.30 (95% CI 0.89 to 1.89), compared to the control group provided with education. In Harrison et al.’s study [36], the effect size of the experimental group provided with web-based education was 0.97 (95% CI 0.93 to 1.01), compared to the control group provided with a pharmacist-led program. In Reese et al.’s study [28], the effect size of the experimental group provided with automated medication reminders was 0.91 (95% CI 0.72 to 1.16), compared to the control group provided with a wireless pill bottle without an alarm. In Suhling et al.’s study [21], the effect size of the experimental group provided with tablet/PC-based education was 0.00 (95% CI -0.49 to 0.49), compared to the control group provided with counseling from a trained nurse.

#### Medication knowledge

Two studies [21, 36] measured medication knowledge. One study [36] used the change in knowledge score measured at three months after the start of the intervention, and the other study [21] used the proportion of participants with improved knowledge after six months. The effect size at three months was 0.24 (95% CI -0.03 to 0.51, n = 1, *P* = .09), which showed no difference from conventional care. The effect size at six months was 1.00 (95% CI 0.15 to 6.67, n = 1, *P* > .99), which was similar to that of conventional care.

## Discussion

### Summary of evidence

To the best of our knowledge, our study is among the first to evaluate the effects of eHealth interventions for improving medication adherence among transplant patients. This systematic review included only RCTs with a high level of evidence among interventional studies. The results of our systematic review and meta-analysis provide evidence of the usefulness of eHealth interventions as a means of improving the quality of clinical practice as well as guidance regarding the development of eHealth interventions that will be effective in increasing medication adherence.

The seven RCTs included in this study were significantly heterogeneous in terms of measurement and data type. Measurement type was divided into objective and subjective medication adherence, and data type was divided into dichotomous and continuous data. Therefore, this study had limitations in combining all seven studies for a pooled estimate, and sub-analysis was conducted by dividing objective and subjective measurement and dichotomous and continuous data. However, except for the sub-analysis of studies that objectively measured medication adherence and presented the results as dichotomous data, the statistical heterogeneity was greater than 80%, or only one study was subject to analysis. Thus, these studies were conducted with narrative synthesis. The meta-analysis demonstrated that the effect size of eHealth interventions for medication adherence was not at all significant, with the effects comparable to those reported for the care provided to the control group. This result differs from the results of a meta-analytic study that reviewed eHealth interventions administered to asthmatic (effect size: 0.41, *P* = .04) [38] or cardiovascular (effect size: 4.51, *P* < .001) [39] patients, in which significant effects were shown. A small number of studies included in this meta-analysis may have resulted in an insignificant analysis of the effects.

Previous systematic reviews considered various interventions and analyzed their effects on improving medication adherence in organ transplant patients [24–26], and most of the studies included in these systematic reviews reported that the interventions were effective [24–26]. These results should be considered in conjunction with the similar effects of eHealth and conventional interventions in this study, which indicate that eHealth interventions are highly likely to be used in the future. Considering that transplant patients need to exercise continuous diligent adherence to medication regimens, as do patients with cardiovascular disease [39] or asthma [38], eHealth interventions for improving medication adherence in transplant patients are potentially valuable for improving medication adherence in general.

The results of the meta-analysis showed no significant differences in the medication adherence of the group provided with eHealth interventions and the control group; however, the individual results of the studies differed. Based on individual studies included in this meta-analysis, the control group for eHealth interventions with a higher effect size was a group that was provided a one-time educational intervention that could occur in standard care. The other eHealth interventions that were not as effective were compared to advanced interventions, such as conducting a pharmacist-led program or using a wireless pill container. In other words, eHealth interventions have a similar effect size to advanced interventions and a higher effect size than standard care. It is also worth noting that eHealth interventions have the advantage of being less labor-intensive for medical staff and have high temporal and spatial accessibility.

eHealth interventions can be used as convenient tools for medication adherence because of their portability and accessibility, which allow for alarms at medication times and make it easy for patients to check to see if they missed a dose. eHealth interventions for improving medication adherence in transplant patients are potentially valuable as interventions to improve medication adherence in general. Further, the likelihood of using eHealth interventions is increasing, due to the rapid development of information and communication technology and the increasing use of mobile phone applications. Considering these rapidly advancing technologies, our study results can be seen as timely and relevant.

There were seven studies [19, 21, 22, 28, 29, 36, 37] in which quantitative or narrative synthesis was conducted to analyze intervention effects for medication adherence. The interventions provided in these studies were mobile applications for self-management [22], mobile application for medication management [29], web-based education [36], mobile phone-based medication monitoring [37], automated medication reminders with wireless pill bottles and physician notifications [28], mobile phone-based spirometry monitoring [19], and tablet/PC-based education [21]. The integration of these interventions showed that education, self-recording and monitoring, reminders, and medical staff monitoring were provided. Education was provided through videos using tablets/PCs, websites, or mobile applications, and self-recordings allowed patients to enter the health indicators throughout the day and easily check their condition through a graphic display function. Reminders using lights, sounds, and messages were provided so that medication times would not be missed. When patients’ health status worsened or medication adherence decreased, this data were sent to medical staff, and the medical staff then contacted the patients to provide feedback. It would be helpful to reference these interventions when creating and developing eHealth interventions in the future.

Two studies measured the effect of eHealth interventions on medication knowledge [21, 36]—in one study, the type of intervention was web-based education [36], and for the other, it was tablet/PC-based education [21]. There were no statistically significant differences compared to face-to-face education from medical professionals in either study, suggesting that, at present, web- or computer-based education has a similar effect to face-to-face education from medical professionals in improving medication knowledge. The results of a previous meta-analysis [40] of conventional interventions provided for the improvement of the medication adherence confirmed significant effects on patients’ medication knowledge. Most of the interventions included in the meta-analysis [40] provided face-to-face intervention and were conducted by providing education or written material to patients. In other words, the effectiveness of eHealth interventions in this meta-analysis was similar to the effectiveness of conventional interventions, indicating it is worthwhile to apply eHealth interventions. Conventional interventions, such as face-to-face methods, take much time and effort to employ. Therefore, the availability of eHealth interventions is likely to increase in the future.

Although medication knowledge and adherence are not directly related, a high level of medication knowledge increases the probability of correctly adhering to one’s medication regimen [40]. In this meta-analysis, only two studies were included to analyze the effects on medication knowledge. Therefore, there are limitations in interpreting the results of the meta-analysis, and more research should be conducted on this issue in the future.

The strengths of this study are as follows. First, it is the first study to analyze the effects of eHealth intervention to improve medication adherence in organ transplantation patients. Second, 11 core databases and standard databases, based on the COSI model [41], were selected to perform a literature search to reduce the bias of literature selection.

### Limitations

Several study limitations should be acknowledged. First, the number of included studies was too small to lead to confirm conclusions. Second, although we tried to include the maximum number of papers in this meta-analysis, there is a possible limitation in that only the studies retrieved through databases were selected, and gray papers, including unpublished studies or theses, were not included. Third, in the results of the ROB assessment for the seven RCTs that were included in the final analysis in this study, less than 50% of the studies showed a low ROB in allocation concealment, blinding of participants and assessors, and blinding for outcome assessment, and the quality of RCTs included in the assessment was not high. The lack of blinding might have resulted in an overestimation of the effects of the resulting variables. Fourth, in this study, when eHealth interventions were provided to patients with organ transplantation, the clinical outcomes of the participants could not be confirmed; instead, we could only confirm the extent of medication adherence and knowledge. Meta-analyses of the clinical outcomes of transplant patients are needed in the future. Fifth, although this study applied a random effect model in consideration of the clinical situation and statistical heterogeneity of the interventions, the effect size could be overestimated by applying more weight to small studies. Sixth, among the studies included in the meta-analysis, the HHS used in DeVito Dabbs’ study [22] is an instrument to measure various self-monitoring behaviors as well as medication adherence. For the meta-analysis, the present study intended to use only results of medication adherence, but because there was a limit in identifying the details in the HHS, the results of the entire instrument were used.

## Conclusions

Meta-analysis and narrative synthesis showed that eHealth interventions for improving medication adherence conducted with organ transplant patients had a similar effect in improving their medication adherence and knowledge compared to standard care or advanced interventions. Therefore, eHealth interventions can be used for medication adherence in organ transplant patients. We recommend further development of eHealth intervention applications, so they may include more features for medication education, self-recording and monitoring, reminders using signals, and monitoring by medical staff to check participants’ health indicators or medication adherence. Further high-quality studies that assess the effects of eHealth interventions for improving medication adherence in organ transplant patients should be conducted to provide support for effective interventions. Additionally, there is a need for standardized measurements and definitions of medication adherence to improve the quality of research in this area.

## Supporting information

S1 Appendix International prospective register of systematic review. (PDF)

S2 Appendix PRISMA checklist. (PDF)

S3 Appendix Example search strategy. (PDF)

S4 Appendix List of studies included in the systematic review. (PDF)
