# Supplementary material for: Effectiveness of eHealth interventions for improving medication adherence of organ transplant patients: A systematic review and meta-analysis
Source: PLoS One. 2020 Nov 5;15(11):e0241857. doi: 10.1371/journal.pone.0241857 (PMC7644069; doi:10.1371/journal.pone.0241857)
Supplement: S3 Appendix — (PDF) [file pone.0241857.s003.pdf]

S3 Appendix. Example search strategy

1) MEDLINE via Pubmed

1. transplantation [MeSH Terms] OR transplantation [Text Word] OR transplant* [Text Word]
2. ehealth [Text Word] OR mhealth [Text Word] OR “e health” [Text Word] OR “m health” [Text Word] OR mobile health [Text Word] OR smartphone [Text Word] OR “smart phone” [Text Word] OR “cell phone” [Text Word] OR “mobile based” [Text Word] OR phone app [Text Word] OR mobile app [Text Word] OR smartphone app [Text Word] OR mobile application [Text Word] OR internet [Text Word] OR smartphone [MeSH Terms] OR cell phone [MeSH Terms] OR mobile applications [MeSH Terms] OR internet [MeSH Terms] OR cellular network [Text Word] OR self care [MeSH] OR self care [Text Word] OR self-management [MeSH Terms] OR self management [Text Word]
3. (medication adherence [Text Word] OR drug adherence [Text Word] medication adherence [MeSH Terms] OR patient compliance [MeSH Terms] OR ((patient compliance [MeSH Terms] AND (drug OR medication)) OR patient medication knowledge [MeSH Terms] OR medication knowledge [Text Word] OR drug knowledge [Text Word] OR compliance [MeSH Terms] OR compliance [Text Word] OR adherence [Text Word])
4. randomized controlled trial [MeSH Terms] OR randomized controlled trial [Text Word] OR randomized controlled study [Text Word]
5. #1 AND #2 AND #3 AND #4
