# Supplementary material for: Effectiveness of eHealth interventions for improving medication adherence of organ transplant patients: A systematic review and meta-analysis
Source: PLoS One. 2020 Nov 5;15(11):e0241857. doi: 10.1371/journal.pone.0241857 (PMC7644069; doi:10.1371/journal.pone.0241857)
Supplement: S4 Appendix — (PDF) [file pone.0241857.s004.pdf]

S4 Appendix. List of studies included in the systematic review

1. Sengpiel J, Fuehner T, Kugler C, Avsar M, Bodmann I, Boemke A, et al. Use of telehealth technology for home spirometry after lung transplantation: A randomized controlled trial. Prog Transplant.2010;20(4):310-7. https://doi.org/10.7182/prtr.20.4.5522u13010202761 PMID: 21265282
2. Suhling H, Rademacher J, Zinowsky I, Fuge J, Greer M, Warnecke G, et al. Conventional vs. tablet computer-based patient education following lung transplantation: A randomized controlled trial. PLoS One.2014;9(3):e90828. https://doi.org/10.1371/journal.pone.0090828 PMID: 24608864
3. DeVito Dabbs A, Song MK, Myers BA, Li R, Hawkins RP, Pilewski JM, et al. A randomized controlled trial of a mobile health intervention to promote self-management after lung transplantation. Am J Transplant.2016;16(7):2172-80. https://doi.org/10.1111/ajt.13701 PMID: 26729617
4. Reese PP, Bloom RD, Trofe-Clark J, Mussell A, Leidy D, Levsky S, et al. Automated reminders and physician notification to promote immunosuppression adherence among kidney transplant recipients: A randomized trial. Am J Kidney Dis.2017;69(3):400-9. https://doi.org/10.1053/j.ajkd.2016.10.017 PMID: 27940063
5. Han A, Min SI, Ahn S, Min SK, Hong HJ, Han N, et al. Mobile medication manager application to improve adherence with immunosuppressive therapy in renal transplant recipients: a randomized controlled trial. PloS ONE. 2019;14(11):e0224595. https://doi.org/10.1371/journal.pone.0224595 PMID: 31689320
6. Harrison JJ, Badr S, Hamandi B, Kim SJ. Randomized controlled trial of a computer-based education program in the home for solid organ transplant recipients: Impact on

medication knowledge, satisfaction, and adherence. Transplantation. 2017;101(6):1336-43. https://doi.org/10.1097/tp.0000000000001279 PMID: 27367473

7. McGillicuddy JW, Gregoski MJ, Weiland AK, Rock RA, Brunner-Jackson BM, Patel SK, et al. Mobile health medication adherence and blood pressure control in renal transplant recipients: a proof-of-concept randomized controled trial. JMIR Res Protoc. 2013;2(2):e32. https://doi.org/10.2196/resprot.2633 PMID: 24004517
