# Supplementary material for: Effectiveness of eHealth interventions for improving medication adherence of organ transplant patients: A systematic review and meta-analysis
Source: PLoS One. 2020 Nov 5;15(11):e0241857. doi: 10.1371/journal.pone.0241857 (PMC7644069; doi:10.1371/journal.pone.0241857)
Supplement: Attachment — Submitted filename: L.Mellon PLoS One review.docx [file pone.0241857.s005.docx]

This study seeks to answer an important research question, whether eHealth interventions can improve medication adherence post organ transplant. Given that non-adherence to the immunosuppressant regimens is one of the most common reasons for graft failure, identifying effective interventions to improve non-adherence is paramount to long term graft survival. eHealth interventions provide a potential inexpensive and interactive way to improve adherence, so testing their efficacy is a timely objective. In this systematic review and meta-analysis, the authors conclude that eHealth interventions have similar effects to conventional care, and are a convenient way to improve medication adherence. The methods and assessment of risk of bias appear sound, however there are significant concerns in the analysis, mainly around 4 issues – 1) The assessment of medication adherence; 2) The definitions of outcomes; 3) The appropriateness of the use of meta-analysis; 4) narrow search terms – some new/key studies missing. These issues lead to the conclusion that the authors have applied meta-analysis in the wrong context here, and that narrative synthesis may be a more suitable method for analysing the data from the 5 included studies. Based on this, my recommendation is that major revisions should be applied to answer the specified research question in the most appropriate manner. Please see below for further points to consider when revising the paper.

**Abstract:**

**LINE 45:** High risk of bias >50% - it is unusual to categorise Risk of Bias in a %, perhaps it may be more useful to highlight which areas within the ROB assessment showed high ROB?

**LINE 50:** The Results section of the abstract reports no difference between intervention and control – is it misleading to then say in Conclusions that eHealth interventions are shown to be viable, convenient and portable and easily accessible? Furthermore, this is a deviation from the specified research question, namely to assess the effect of an eHealth intervention – it isn’t mentioned that the aim was to assess the viability, accessibility, portability or convenience, so perhaps re-word this to better reflect the primary research question.

**Introduction**

**Lines 81-83:** References #9,10,11 give good figures for rates of non-adherence. Work by Dew et al (Dew MA, et al. Rates and Risk factors for nonadherence to the medical regimen after adult solid organ transplantation. Transplantation 2007;83(7):858-73. [MEDLINE: 17460556]) and Denhaerynck (Denhaerynck K, et al. Prevalence, consequences, and determinants of nonadherence in adult renal transplant patients: a literature review. Transplant International 2005;18(10):1211-33. [MEDLINE: 16162098]) also provide good estimates from pooled data across all organ types, and may be useful references.

**Lines 90-91**: A definition of what constitutes an eHealth intervention for the purposes of this study is needed here. In general, there is significant variability in the content/type of eHealth interventions used internationally. Not all apps have feedback and counselling in real time, and some are nothing more than simple reminders to take medication. In the 5 studies included in this review, one is a web-based educational intervention (Harrison 2017), and web delivered interventions aren’t listed here in the Introduction as types of eHealth.

**Line 97-98**: This sentence is unclear, do the authors mean to say “analyse the effects of medication adherence *interventions?”*

**Line 106-107**: It’s unclear what the authors mean by “present the direction of development of eHealth intervention for organ transplant recipients”, and how this is related to the meta-analysis? Clarity/rewording of this sentence would be useful.

**Methods**

**Line 114**: The last search was August 2019 – this is a rapidly evolving area and some key interventions were published in 2019/2020, perhaps it may be useful to update the search? There are also some studies pre-2019 that may fit the study inclusion criteria, and involve similar eHealth interventions to that which are included in the final analysis below. See below for examples of studies (pre/post 2019) that may fit:

- Hardstaff R, Green K, Talbot D. Measurement of compliance posttransplantation--the results of a 12-month study using electronic monitoring. Transplantation Proceedings 2003;35(2):796-7. [MEDLINE: 12644142] 🡪 *Similar intervention to Reese 2017*
- Henriksson J, Tyden G, Hoijer J, Wadstrom J. A prospective randomized trial on the effect of using an electronic monitoring drug dispensing device to improve adherence and compliance. Transplantation 2016;100(1):203-9. [MEDLINE: 26588006] 🡪 *Similar intervention to Reese 2017, a web-based platform was used to provide feedback*
- Korus M, Cruchley E, Calic M, Gold A, Anthony SJ, Parekh RS, et al. Assessing the acceptability and efficacy of teens taking charge: Transplant-A pilot randomized control trial. Pediatric Transplantation 2020;24(1):e13612. [MEDLINE: 31743564] 🡪 *is listed as a pilot study, but has similar sample size to studies included in the review*
- McGillicuddy JW, Gregoski MJ, Weiland AK, Rock RA, Brunner-Jackson BM, Patel SK, et al. Mobile health medication adherence and blood pressure control in renal transplant recipients: a proof-of-concept randomized controlled trial. JMIR Research Protocols 2013;2(2):e32. [MEDLINE: 24004517] 🡪 *is listed as a pilot study, but has similar sample size to studies included in the review*
- Han A, Min SI, Ahn S, Min SK, Hong HJ, Han N, et al. Mobile medication manager application to improve adherence with immunosuppressive therapy in renal transplant recipients: a randomized controlled trial. PLoS ONE [Electronic Resource] 2019;14(11):e0224595. [MEDLINE: 31689320]
- Kaier K, Hils S, Fetzer S, Hehn P, Schmid A, Hauschke D, et al. Results of a randomized controlled trial analyzing telemedically supported case management in the first year after living donor kidney transplantation - a budget impact analysis from the healthcare perspective. Health Economics Review 2017;7(1):1. [MEDLINE: 28092012]
- Levine D, Torabi J, Choinski K, Rocca JP, Graham JA. Transplant surgery enters a new era: Increasing immunosuppressive medication adherence through mobile apps and smart watches. American journal of surgery 2019;218(1):18-20. [MEDLINE: 30799019]

**Line 121:** Medication knowledge outcomes are only mentioned here as an outcome, and not described in the Introduction. If this is to be a key focus of the paper, it would be useful to provide the rationale and current evidence for focusing on medication knowledge earlier in the paper.

**Line 141-143:** When assessing medication adherence in chronic diseases in general, there is poor correlation between self-reported adherence and objectively measured adherence (for example: Moran K, et al. The INCA^TM^ (Inhaler Compliance Aid): A comparison with established measures of adherence. Psychology and Health. 2017;32:1266-1287 doi: 10.1080/08870446.2017.1290243.). In the literature regarding medication adherence post-transplant, self-reported medication adherence is poorly associated with clinical outcomes (for example, Scheel J, et al. BMC Nephrology. 2017:18;107 DOI: <https://doi.org/10.1186/s12882-017-0517-6>), and it is argued that the most accurate way to measure adherence is to include both objective adherence (via clinical measures, such as tacrolimus serum concentration levels, pill counts, prescription refill data) and subjective measures (e.g. self-report via instruments), as this then accounts for both intentional and non-intentional adherence. It seems counterintuitive then to focus only on self-reported adherence via instruments, as stated here. A stronger rationale is required for only including self-reported instruments of medication adherence in the review.

Further to this, the authors state on **Line 143** that only studies using self-report (instruments) will be examined, however the meta-analysis presented in the Results section includes 2 studies that have measured adherence through objective means only (Sengpiel, Suhling).

**Line 143:** The HHS, BAASIS and MACS are mentioned as examples of self-reported adherence instruments. Were studies excluded if they used other self-report measures, for example the MMAS? Were any specific scales for medication knowledge identified prior to the study?

A comment on these measures – the HHS, which is used in the RCT by DeVito Dabbs, is a measure that includes more than just medication adherence. It measures a range of self-monitoring behaviours, not just medication adherence, so including this as a total score in meta-analysis as a measure representing medication adherence isn’t accurate.

**Line 149**: Why were pilot studies excluded? A stronger rationale for excluding pilot studies is required, particularly as there as good quality trials in this area that are classed as pilot studies, and have similar sample sizes to the 5 included studies.

**Line 182:** Would it be useful to include sub-group analysis for types of eHealth intervention, given the variability across eHealth interventions?

**Line 184**: “And total and events”. It would be useful if the authors could clarify what is meant by total and events? In addition to this, a definition of what constitutes non-adherence in each instrument would be useful, as there is significant variation across measures in defining non-adherence.

**Results:**

*Included studies:*

- Harrison et al. is a web-based intervention – this isn’t mentioned in the author’s discussion of eHealth earlier in Introduction, therefore a definition of eHealth is necessary as a wide range of interventions are included. For Harrison, the MAC is used - this measure includes a combination of both objective and subjective scores to give an overall adherence score, therefore can authors accurately extract the self-report data from the MAC?
- Sengpiel et al. I’m unclear why this study was included, as adherence is not measured by an instrument, and therefore violates the study inclusion criteria outlined in Methods?
- Suhling et al. – is this an educational intervention delivered via electronic means, or an eHealth intervention? Again, adherence is only measured by an objective outcome, so I’m unclear why this study was included.

**Line 244/245**: “Medication adherence was divided into two types depending on the cut point”. Was this a cut point per instrument, or an overall cut point? See above re. the need for a definition of non-adherence.

**Table 2** -**Part A**: it is unclear what data is being presented here? The effect size from the meta-analysis is presented in the top row under dichotomous data, but includes both dichotomous and continuous data, from both objective and subjective measures of medication adherence.

**Table 2 - Part B**: It isn’t clear what these data refer to.

**Line 276 onwards:** I have concerns about the use of meta-analysis in this instance. The 5 included studies are all combined for a pooled estimate, despite significant heterogeneity in terms of type of measurement (objective adherence and subjective adherence) and heterogeneity of measurement (dichotomous versus continuous data). The pooled estimate is subsequently flawed, and doesn’t accurately answer the research question.

Even amongst the three studies that use self-reported measures, there is heterogeneity between measures, so I’m unsure how confident authors can be in the reliability or accuracy of the outcome of the meta-analysis. For example, Harrison et al. present both self-report and objective data as part of the MACS measure, so how was this entered?

In the forest plot, studies are pooled that are all measuring very different things. The rationale behind pooling all five data sets would need clear explanation.

**Discussion**

General comment: the conclusions may be overreaching based on the above concerns regarding the accurateness of the presented meta-analysis results.

**Line 408** – It is mentioned here that ROB assessment was completed for seven studies, however only 5 studies are included in analysis. Perhaps this is a typo for correction.

**Appendices:**

**Flowchart:** Bottom box reports 5 studies included in qualitative synthesis – should this say five studies included in meta-analysis?

**Forest plot:** See above for concerns regarding suitability of meta-analysis in this instance.
