# Supplementary material for: Effectiveness of eHealth interventions for improving medication adherence of organ transplant patients: A systematic review and meta-analysis
Source: PLoS One. 2020 Nov 5;15(11):e0241857. doi: 10.1371/journal.pone.0241857 (PMC7644069; doi:10.1371/journal.pone.0241857)
Supplement: Attachment — Submitted filename: Response to reviewers.docx [file pone.0241857.s006.docx]

**Response to the review report**

Thank you for your valuable comments on our manuscript entitled “Effectiveness of eHealth interventions for improving medication adherence of organ transplant patients: A systematic review and meta-analysis.” After we revised the manuscript in a point-by-point manner following the reviewers’ comments, we changed the edited sentences to red font. We have made an earnest effort to revise the manuscript in accordance with the reviewers’ suggestions. Please see our responses to the individual comments below.

**Reviewer 1.**

This study seeks to answer an important research question, whether eHealth interventions can improve medication adherence post organ transplant. Given that non-adherence to the immunosuppressant regimens is one of the most common reasons for graft failure, identifying effective interventions to improve non-adherence is paramount to long term graft survival. eHealth interventions provide a potential inexpensive and interactive way to improve adherence, so testing their efficacy is a timely objective. In this systematic review and meta-analysis, the authors conclude that eHealth interventions have similar effects to conventional care, and are a convenient way to improve medication adherence. The methods and assessment of risk of bias appear sound, however there are significant concerns in the analysis, mainly around 4 issues – 1) The assessment of medication adherence; 2) The definitions of outcomes; 3) The appropriateness of the use of meta-analysis; 4) narrow search terms – some new/key studies missing. These issues lead to the conclusion that the authors have applied meta-analysis in the wrong context here, and that narrative synthesis may be a more suitable method for analysing the data from the 5 included studies. Based on this, my recommendation is that major revisions should be applied to answer the specified research question in the most appropriate manner. Please see below for further points to consider when revising the paper.

Answer) Thank you for your comment. we reran the meta-analysis by separating the studies between those with objective and subjective measurement and those with dichotomous and continuous data. Although the search term was not changed, the literature search conducted allowed us to add the more recent study and one new study by changing the search exclusion criteria (allowing inclusion of pilot studies).

**Abstract**

1. LINE 45: High risk of bias >50% - it is unusual to categorise Risk of Bias in a %, perhaps it may be more useful to highlight which areas within the ROB assessment showed high ROB?

Answer) We changed the sentence to emphasize which part represented a high ROB. Five studies explained that ROB was high in the blinding of participants and personnel areas.

2. LINE 50: The Results section of the abstract reports no difference between intervention and control – is it misleading to then say in Conclusions that eHealth interventions are shown to be viable, convenient and portable and easily accessible? Furthermore, this is a deviation from the specified research question, namely to assess the effect of an eHealth intervention – it isn’t mentioned that the aim was to assess the viability, accessibility, portability or convenience, so perhaps re-word this to better reflect the primary research question.

Answer) Thank you for your comment. We have confirmed that there is a deviation in the research questions and conclusions. The research questions were intended to assess the effectiveness of eHealth interventions. Thus, we deleted sentences that were not related to the focus of the study. The edit is as follows:

“Our findings suggest that eHealth interventions were similar to standard care or advanced care for improving medication adherence, and they faired equally well for improving medication knowledge. Therefore, eHealth intervention can be used for medication adherence of organ transplant patients.”

**Introduction**

3. Lines 81-83: References #9,10,11 give good figures for rates of non-adherence. Work by Dew et al (Dew MA, et al. Rates and Risk factors for nonadherence to the medical regimen after adult solid organ transplantation. Transplantation 2007;83(7):858-73. [MEDLINE: 17460556]) and Denhaerynck (Denhaerynck K, et al. Prevalence, consequences, and determinants of nonadherence in adult renal transplant patients: a literature review. Transplant International 2005;18(10):1211-33. [MEDLINE: 16162098]) also provide good estimates from pooled data across all organ types, and may be useful references.

Answer) We have added evidence for the rate of medication non-adherence by quoting the results of your recommended citations, including Dew et al. Thanks to your comments, more research results have been presented.

4. Lines 90-91: A definition of what constitutes an eHealth intervention for the purposes of this study is needed here. In general, there is significant variability in the content/type of eHealth interventions used internationally. Not all apps have feedback and counselling in real time, and some are nothing more than simple reminders to take medication. In the 5 studies included in this review, one is a web-based educational intervention (Harrison 2017), and web delivered interventions aren’t listed here in the Introduction as types of eHealth.

Answer) We have redefined eHealth intervention, and the contents are as follows.

“eHealth-based interventions employ electronic processes and communication through the use of electronic, computer, or mobile devices [16,17]. The information provided can include health education information; monitoring, recording, and transmitting of data about health behaviors and health indicators, such as blood pressure or blood sugar levels; and providing reminders, feedback, and counseling to patients through mobile apps, the Internet, and electronic devices [16,17].” (page 5).

5. Line 97-98: This sentence is unclear, do the authors mean to say “analyse the effects of medication adherence interventions?”

Answer) We have changed this sentence for clarity as follows:

“…it is necessary to comprehensively analyze the effects of eHealth interventions for medication adherence…” (page 5)

6. Line 106-107: It’s unclear what the authors mean by “present the direction of development of eHealth intervention for organ transplant recipients”, and how this is related to the meta-analysis? Clarity/rewording of this sentence would be useful.

Answer) We have changed this sentence for clarity to suggest how eHealth intervention for organ transplant recipients is effectively organized.

**Methods**

7. Line 114: The last search was August 2019 – this is a rapidly evolving area and some key interventions were published in 2019/2020, perhaps it may be useful to update the search? There are also some studies pre-2019 that may fit the study inclusion criteria, and involve similar eHealth interventions to that which are included in the final analysis below. See below for examples of studies (pre/post 2019) that may fit:

- Hardstaff R, Green K, Talbot D. Measurement of compliance posttransplantation--the results of a 12-month study using electronic monitoring. Transplantation Proceedings 2003;35(2):796-7. [MEDLINE: 12644142] 🡪 *Similar intervention to Reese 2017*
- Henriksson J, Tyden G, Hoijer J, Wadstrom J. A prospective randomized trial on the effect of using an electronic monitoring drug dispensing device to improve adherence and compliance. Transplantation 2016;100(1):203-9. [MEDLINE: 26588006] 🡪 *Similar intervention to Reese 2017, a web-based platform was used to provide feedback*
- Korus M, Cruchley E, Calic M, Gold A, Anthony SJ, Parekh RS, et al. Assessing the acceptability and efficacy of teens taking charge: Transplant-A pilot randomized control trial. Pediatric Transplantation 2020;24(1):e13612. [MEDLINE: 31743564] 🡪 *is listed as a pilot study, but has similar sample size to studies included in the review*
- McGillicuddy JW, Gregoski MJ, Weiland AK, Rock RA, Brunner-Jackson BM, Patel SK, et al. Mobile health medication adherence and blood pressure control in renal transplant recipients: a proof-of-concept randomized controlled trial. JMIR Research Protocols 2013;2(2):e32. [MEDLINE: 24004517] 🡪 *is listed as a pilot study, but has similar sample size to studies included in the review*
- Han A, Min SI, Ahn S, Min SK, Hong HJ, Han N, et al. Mobile medication manager application to improve adherence with immunosuppressive therapy in renal transplant recipients: a randomized controlled trial. PLoS ONE [Electronic Resource] 2019;14(11):e0224595. [MEDLINE: 31689320]
- Kaier K, Hils S, Fetzer S, Hehn P, Schmid A, Hauschke D, et al. Results of a randomized controlled trial analyzing telemedically supported case management in the first year after living donor kidney transplantation - a budget impact analysis from the healthcare perspective. Health Economics Review 2017;7(1):1. [MEDLINE: 28092012]
- Levine D, Torabi J, Choinski K, Rocca JP, Graham JA. Transplant surgery enters a new era: Increasing immunosuppressive medication adherence through mobile apps and smart watches. American journal of surgery 2019;218(1):18-20. [MEDLINE: 30799019]

Answer) We updated the results by re-running the meta-analysis on April 17, 2020. Among the studies presented as examples, those by McGillicuddy et al. and Han et al. were included in the meta-analysis. The reasons for not including the rest of the studies are as follows: Hardstaff’s study did not present an exact time point for measuring medication adherence or exact criteria for medication adherence. Henriksson’s study did not measure the medication adherence of the control group. In Korus’ study, the participants were not adults. Keier’s research is related to medical service costs, and Levine’s study shows only the immunosuppressants blood concentration of the experimental group and the control group, it does not present the exact proportion of participant to adherence the medication.

8. Line 121: Medication knowledge outcomes are only mentioned here as an outcome, and not described in the Introduction. If this is to be a key focus of the paper, it would be useful to provide the rationale and current evidence for focusing on medication knowledge earlier in the paper.

Answer) Thank you for your important comment. We have added some information on knowledge of the medication to the introduction. The additions are as follows:

“Recipients of organ transplants are required to take lifelong immunosuppressants after discharge, so they must have adequate knowledge of the medication. The higher the knowledge level, the better the patient’s adherence of medication and treatment instructions and the better the outcome of treatment [13]. Previously, discharge education was mainly provided to improve the knowledge of organ transplant patients [14]” (page 5).

9. Line 141-143: When assessing medication adherence in chronic diseases in general, there is poor correlation between self-reported adherence and objectively measured adherence (for example: Moran K, et al. The INCATM (Inhaler Compliance Aid): A comparison with established measures of adherence. Psychology and Health. 2017;32:1266-1287 doi: 10.1080/08870446.2017.1290243.). In the literature regarding medication adherence post-transplant, self-reported medication adherence is poorly associated with clinical outcomes (for example, Scheel J, et al. BMC Nephrology. 2017:18;107 DOI: https://doi.org/10.1186/s12882-017-0517-6), and it is argued that the most accurate way to measure adherence is to include both objective adherence (via clinical measures, such as tacrolimus serum concentration levels, pill counts, prescription refill data) and subjective measures (e.g. self-report via instruments), as this then accounts for both intentional and non-intentional adherence. It seems counterintuitive then to focus only on self-reported adherence via instruments, as stated here. A stronger rationale is required for only including self-reported instruments of medication adherence in the review.

Further to this, the authors state on Line 143 that only studies using self-report (instruments) will be examined, however the meta-analysis presented in the Results section includes 2 studies that have measured adherence through objective means only (Sengpiel, Suhling).

Answer) We apologize for the misleading content in the text. This meta-analysis analyzed both objective and subjective measurements. Therefore, the following changes were made for clarity.

“As for outcomes, we only included medication adherence and medication knowledge outcome measurements from studies involving eHealth interventions for transplant recipients as objective measures (via clinical measures such as Tacrolimus serum concentration levels, pill counts, and prescription refill data) and subjective measures (e.g., self-reported questionnaires such as the Health Habits Survey [22], Basel Assessment of Adherence to Immunosuppressive Medication Scale [29,30], and Multidimensional Adherence Classification System [31])” (page 8).

We reconducted the meta-analysis, dividing the studies into those using objective measurement and those using subjective measurement.

10. Line 143: The HHS, BAASIS and MACS are mentioned as examples of self-reported adherence instruments. Were studies excluded if they used other self-report measures, for example the MMAS? Were any specific scales for medication knowledge identified prior to the study?

A comment on these measures – the HHS, which is used in the RCT by DeVito Dabbs, is a measure that includes more than just medication adherence. It measures a range of self-monitoring behaviours, not just medication adherence, so including this as a total score in meta-analysis as a measure representing medication adherence isn’t accurate.

Answer) This meta-analysis includes all studies using instruments that measure medication adherence. It was not possible to extract only the parts of the composition of the HHS that measured medication adherence. Therefore, we used the results measured using the entire HHS. This is now described in the limitations of the study (page 26).

11. Line 149: Why were pilot studies excluded? A stronger rationale for excluding pilot studies is required, particularly as there as good quality trials in this area that are classed as pilot studies, and have similar sample sizes to the 5 included studies.

Answer) Thank you for your valuable comments. We removed pilot studies from the exclusion criteria. Among pilot studies, those that met the selection criteria were included in the meta-analysis. The added study is McGillicuddy JW, Gregoski MJ, Weiland AK, Rock RA, Brunner-Jackson BM, Patel SK, et al. Mobile health medication adherence and blood pressure control in renal transplant recipients: a proof-of-concept randomized controlled trial. JMIR Research Protocols 2013;2(2):e32. [MEDLINE: 24004517]

12. Line 182: Would it be useful to include sub-group analysis for types of eHealth intervention, given the variability across eHealth interventions?

Answer) We tried to analyze sub-groups according to the type of intervention. However, each sample size was too small when it was divided into subgroups according to the type of intervention and classified as objective and subjective measurements. The results of the sub-group analysis of the intervention type were deemed meaningless. In response, the sub-group analysis of the intervention type was not presented in the results.

13. Line 184: “And total and events”. It would be useful if the authors could clarify what is meant by total and events? In addition to this, a definition of what constitutes non-adherence in each instrument would be useful, as there is significant variation across measures in defining non-adherence.

Answer) The meanings of total and events have been described more clearly as follows.

“All outcome measurements were extracted as mean and standard deviation (or transformed) or number of events and total sample size” (page 10).

Furthermore, we explained in detail how each study defined medication adherence (pages 16–17).

**Result**

14. *Included studies:*

- Harrison et al. is a web-based intervention – this isn’t mentioned in the author’s discussion of eHealth earlier in Introduction, therefore a definition of eHealth is necessary as a wide range of interventions are included. For Harrison, the MAC is used - this measure includes a combination of both objective and subjective scores to give an overall adherence score, therefore can authors accurately extract the self-report data from the MAC?

Answer) We have reclarified the definition of eHealth intervention. In addition, objective and subjective scores of MAC were extracted separately and used for meta-analysis. Objective scores used the number of participants whose Tacrolimus blood concentration was 3 below the standard deviation and the subjective scores were the number of participants who reported that the rate of non-adherence was less than 10%.

- Sengpiel et al. I’m unclear why this study was included, as adherence is not measured by an instrument, and therefore violates the study inclusion criteria outlined in Methods?
- Suhling et al. – is this an educational intervention delivered via electronic means, or an eHealth intervention? Again, adherence is only measured by an objective outcome, so I’m unclear why this study was included.

Answer) As mentioned earlier, the inclusion criteria for this study have been clearly redefined to include both objective and subjective measurements. The studies of Sengpiel et al. and Suhling et al. carried out objective measurements such as immunosuppressant blood concentration, and the two studies were included by redefining inclusion criteria.

15. Line 244/245: “Medication adherence was divided into two types depending on the cut point”. Was this a cut point per instrument, or an overall cut point? See above re. the need for a definition of non-adherence.

Answer) The details of the criteria for medication adherence in this study have been described in detail, and they are presented on page 16–17.

16. Table 2 -Part A: it is unclear what data is being presented here? The effect size from the meta-analysis is presented in the top row under dichotomous data, but includes both dichotomous and continuous data, from both objective and subjective measures of medication adherence.

Answer) We identified the characteristics of the instruments used in the seven studies included in the meta-analysis, then divided the studies into objective and subjective results. Meta-analysis was conducted by separating the stories according to the characteristics of the data (dichotomous or continuous), and the results have been presented separately.

17. Table 2 - Part B: It isn’t clear what these data refer to.

Answer) There were two studies that measured medication knowledge. One was expressed as dichotomous data and the other was expressed as continuous data, making it impossible to synthesize them. Therefore, we found each effect size and have presented them in the table. We modified the table by combining part A and part B.

18. Line 276 onwards: I have concerns about the use of meta-analysis in this instance. The 5 included studies are all combined for a pooled estimate, despite significant heterogeneity in terms of type of measurement (objective adherence and subjective adherence) and heterogeneity of measurement (dichotomous versus continuous data). The pooled estimate is subsequently flawed, and doesn’t accurately answer the research question.

Even amongst the three studies that use self-reported measures, there is heterogeneity between measures, so I’m unsure how confident authors can be in the reliability or accuracy of the outcome of the meta-analysis. For example, Harrison et al. present both self-report and objective data as part of the MACS measure, so how was this entered?

In the forest plot, studies are pooled that are all measuring very different things. The rationale behind pooling all five data sets would need clear explanation.

Answer) As mentioned earlier, we reran the meta-analysis by separating the studies between those with objective and subjective measurement and dichotomous and continuous data. The concerns that clinical and statistical heterogeneity could result in overestimated effects have been described as limitations of the study (pages 25–26).

**Discussion**

19. General comment: the conclusions may be overreaching based on the above concerns regarding the accurateness of the presented meta-analysis results.

Answer) The results of the re-implementation of the meta-analysis have been described as clearly as possible. The revised conclusions are as follows.

“Meta-analysis showed that eHealth interventions for improving medication adherence conducted with organ transplant patients had a similar effect in improving their medication adherence and knowledge compared to standard care or advanced intervention. Therefore, eHealth intervention can be used for medication adherence of organ transplant patients.” (page 26)

20. Line 408 – It is mentioned here that ROB assessment was completed for seven studies, however only 5 studies are included in analysis. Perhaps this is a typo for correction.

Answer) As a result of re-implementing the meta-analysis, the final analysis included seven studies, and the ROB assessment for the seven studies has been completed. This has been updated in the manuscript.

**Appendices**

21. Flowchart: Bottom box reports 5 studies included in qualitative synthesis – should this say five studies included in meta-analysis?

Answer) We have updated the manuscript to show that seven studies were included in the meta-analysis.

22. Forest plot: See above for concerns regarding suitability of meta-analysis in this instance.

Answer) We re-analyzed the meta-analysis and have presented a forest plot for all four results.

Thank you for your comments.

**Reviewer 2.**

1. The introduction flow hits a confusing stride in first stating the negatives of immunosuppressant medications, stating they are a major cause of complications and costs to the healthcare system from their side effects, and then reversing that they are life-saving and must be adhered to. While these points are both true, consider reorganizing this to get to the objective of the study and to validate the importance of adherence to these medications.

Answer) Thank you for your important comment. We reconstructed the introduction according to the subject matter of this study, adherence of immunosuppressant. The introduction is outlined below.

Major complications of organ transplants include the rejection of transplants, and immunosuppressant should be taken to prevent them. However, up to 60% of late acute rejection and about 30-35% of graft loss is due to non-adherence. The rate of non-adherence of immunosuppressant is high. Medication knowledge, which is mainly provided through discharge education, is important. Since eHealth intervention is currently developing, it is necessary to determine the effects of medication adherence and medication knowledge through eHealth intervention (pages 4–6).

2. An important consideration that I think was overlooked in the discussion was the fact that you were evaluating eHealth inventions to both what would be considered standard care or to another intervention not involving eHealth. Defining what is standard care versus an actual intervention is a very blurry line that all researchers struggle with and this manuscript should not be rejected due to this. I understand this limitation is due to the availability of literature and how these studies are reported, but it must be discussed more. If you look at the effect sizes based on the individual studies, the two eHealth interventions that showed higher effectiveness were versus what is basically a one-time consultation that would happen in standard care. The other eHealth interventions that were not as effective were compared to what seems to be more advanced interventions involving multiple pharmacist meetings or having a wireless pill container. I think this is really important to note that while these "standard" interventions happening face-to-face may be just as effective as eHealth, their consumption of resources and often times inaccessibility are a reason for advocating for eHealth interventions.

Answer) Thank you; we completely agree with your comment. We have added the following paragraph to the discussion.

“The results of the meta-analysis showed no significant difference in the medication adherence of the group provided with eHealth intervention and the control group, but the individual results of the studies differed. Based on individual studies included in this meta-analysis, the control group for eHealth intervention with a higher effect size was a group that was provided one-time education that could occur in standard care. The other eHealth interventions that were not as effective were compared to advanced interventions, such as conducting a pharmacist-led program or using a wireless pill container. In other words, eHealth intervention has a similar effect size as advanced intervention and has a higher effect size compared to standard care. It is also worth noting that it has the advantage of being less labor-intensive for medical staff and has high temporal and spatial accessibility” (page 23).

3. The I squared of 0 is very biased due to their only being five studies included. This is an important limitation and consideration in the heterogeneity being discussed. I do appreciate the detail on the measures of adherence used in all studies. If I understand correctly, three used a questionnaire and two used drug concentration? While both valid measures of adherence, they would differ considerably.

Answer) We identified the characteristics of the instruments used in the seven studies included in the meta-analysis, then divided them into objective and subjective studies. The meta-analysis was conducted by separating them according to the characteristics of the data (dichotomous or continuous), and the results have been presented separately.

As a result of rerunning the meta-analysis, the results of statistical heterogeneity as well as clinical heterogeneity were thoroughly measured. The limitations of the synthesis of these heterogeneous studies are described as limitations of this study.

Thank you for your comments.
