# Supplementary material for: Effectiveness of eHealth interventions for improving medication adherence of organ transplant patients: A systematic review and meta-analysis
Source: PLoS One. 2020 Nov 5;15(11):e0241857. doi: 10.1371/journal.pone.0241857 (PMC7644069; doi:10.1371/journal.pone.0241857)
Supplement: Attachment — Submitted filename: Response to reviewers.docx [file pone.0241857.s007.docx]

**Response to the review report**

Thank you for your valuable comments regarding our manuscript entitled “Effectiveness of eHealth interventions for improving medication adherence of organ transplant patients: A systematic review and meta-analysis.” We carried out a point-by-point revision of the manuscript following the academic editor and reviewers’ comments, and indicated the edited sentences in red font. We have made an earnest effort to revise the manuscript in accordance with the academic editor and reviewers’ suggestions. Please see our responses to the individual comments below.

**Academic editor**

Some meta-analysis were performed, despite statistical significant test for heterogeneity (meta-analysis A and D). In its current form the analysis adequate. The authors should either only perform a structured narrative synthesis or perform an in depth analysis of heterogeneity in these cases.

Response: Thank you for your comment. Meta-analyses A, B, and D were changed to narrative synthesis. Meta-analysis C, however, was not changed, as statistical heterogeneity was found to be 50%. Additionally, Figure A was changed to Figure 5, Figure B to Figure 6, Figure C to Figure 3, and Figure D to Figure 4. The study results were modified accordingly (lines 336–403).

please structure the manuscript as suggested in PRISMA (e.g. the section on risk of bias before the results).

Response: The paper’s overall structure was changed to follow the order suggested by PRISMA.

**Reviewer 1**

The authors have made significant amendments to the paper that have strengthened it considerably. I still disagree however with the use of meta-analysis, given the significant heterogeneity across the measures used here. The authors have given their rationale for proceeding with meta-analysis, and therefore I have flagged some concerns where the authors need to provide more information to flag the significant heterogeneity between studies and their method of measurement.

Response: Thank you for your comment. Meta-analyses A, B, and D were changed to narrative synthesis. Meta-analysis C, however, was not changed, as statistical heterogeneity was found to be 50%. Additionally, Figure A was changed to Figure 5, Figure B to Figure 6, Figure C to Figure 3, and Figure D to Figure 4. The study results were modified accordingly (lines 336–403).

1. Page 16: Outcomes section: I think it would be useful to highlight in this section the significant heterogeneity across the studies in how adherence was measured. The authors have provided more information here on how non-adherence was categorised, but it is still vague in parts. For example, how were the tacrolimus blood concentrations levels categorised as non-adherent across studies? Different authors use different algorithms, so it is important to detail the variance in categorisation to readers. Similarly, for the self-reported questionnaires, more detail is required here to explain to readers the heterogeneity across measures.

Response: We emphasized that the measurement methods for medication adherence in the studies included in the meta-analysis were heterogeneous. In each study, the methods for measuring the adherence of medication, such as Tacrolimus blood concentrations levels or self-report questionnaires, were described in detail regarding how adherence was defined (Outcome section, pages 15–17).

2. Table 2: I’m unsure as to what is being presented here for medication knowledge. Given that there was one study with a dichotomous measure of knowledge, and one with a continuous measure, I’m presuming this wasn’t meta-analysed, but as it is in a table with data from meta-analysis for medication adherence, this is misleading. I suggest reporting findings for knowledge in text alone for clarity.

Response: Thank you for your comment. We deleted Table 2 and reported the results on medication knowledge only in the text, as follows:

“Two studies [21,30] measured medication knowledge. One study [30] used the change in knowledge score measured at three months after the start of the intervention, and the other study [21] used the proportion of participants with improved knowledge after six months. The effect size at three months was 0.24 (95% CI -0.03 to 0.51, n = 1, 𝑃 = .09), which showed no difference from conventional care. The effect size at six months was 1.00 (95% CI 0.15 to 6.67, n = 1, 𝑃 > .99), which was similar to that of conventional care.” (page 22)

3. Page 26: The limitations of including the HHS as an overall medication adherence score are addressed, however it isn’t acknowledged that the MACS is also a multidimensional adherence scale and including this as a total score may produce misleading results.

Response: The MACS comprises self-reporting and immunosuppressant blood concentration. Since self-reporting and immunosuppressant blood concentration results were presented separately in the Harrison et al. study, we used these results in the analysis, separating them into subjective and objective data.

4. Line 457: It may be useful to comment that alongside high quality studies, there is a need for standardised measurement and definitions of adherence to improve the quality of the research in this area.

Response: Thank you for your important comment. We added the following sentence at the end of the conclusion:

“Additionally, there is a need for standardized measurement and definitions of medication adherence to improve the quality of research in this area.” (page 28)

5. Figure 2: Figs B & D – I fail to see the usefulness of the statistics presented here in these two estimates of effect size. Fig D, a pooled estimate from 2 studies, with an I2 of 97% is like comparing apples with oranges! It would be more useful to the reader to report this in narrative synthesis. Perhaps Chapter 10 of the Cochrane handbook may provide guidance on the appropriateness of the data for meta-analysis.

Response: Thank you for your comment. As noted above, meta-analyses A, B, and D were changed to narrative synthesis. Meta-analysis C, however, was not changed, as statistical heterogeneity was found to be 50%. Additionally, Figure A was changed to Figure 5, Figure B to Figure 6, Figure C to Figure 3, and Figure D to Figure 4. The study results were modified accordingly (lines 336–403).

6. For figs A-D, it would be useful to add a note to each study in RevMan listing the method of adherence measurement (e.g. name of questionnaire). A title for each is also required – it’s not clear from current figure what C&D represent?

Response: At the bottom of Figures A through D, we specified the medication adherence measurement methods used in each study as footnotes. A, B, C, and D were divided into figures 3, 4, 5, and 6 and titled respectively, clearly describing what data were used for each figure.
