# Supplementary material for: Effectiveness of eHealth interventions for improving medication adherence of organ transplant patients: A systematic review and meta-analysis
Source: PLoS One. 2020 Nov 5;15(11):e0241857. doi: 10.1371/journal.pone.0241857 (PMC7644069; doi:10.1371/journal.pone.0241857)
Supplement: Peer review history

# Peer review: Decision Letter 0

**DOI:** 10.1371/journal.pone.0241857.r001

**Authors:** Tim Mathes

14 Apr 2020

PONE-D-20-07165

Effectiveness of eHealth intervention for improving medication adherence of organ transplant patients: A systematic review and meta-analysis

PLOS ONE

Dear Dr Seo,

Thank you for submitting your manuscript to PLOS ONE. After careful consideration, we feel that it has merit but does not fully meet PLOS ONE’s publication criteria as it currently stands. Therefore, we invite you to submit a revised version of the manuscript that addresses the points raised during the review process.

We would appreciate receiving your revised manuscript by May 29 2020 11:59PM. When you are ready to submit your revision, log on to https://www.editorialmanager.com/pone/ and select the 'Submissions Needing Revision' folder to locate your manuscript file.

If you would like to make changes to your financial disclosure, please include your updated statement in your cover letter.

To enhance the reproducibility of your results, we recommend that if applicable you deposit your laboratory protocols in protocols.io, where a protocol can be assigned its own identifier (DOI) such that it can be cited independently in the future. For instructions see: http://journals.plos.org/plosone/s/submission-guidelines#loc-laboratory-protocols

Please include the following items when submitting your revised manuscript:

- A rebuttal letter that responds to each point raised by the academic editor and reviewer(s). This letter should be uploaded as separate file and labeled 'Response to Reviewers'.
- A marked-up copy of your manuscript that highlights changes made to the original version. This file should be uploaded as separate file and labeled 'Revised Manuscript with Track Changes'.
- An unmarked version of your revised paper without tracked changes. This file should be uploaded as separate file and labeled 'Manuscript'.

Please note while forming your response, if your article is accepted, you may have the opportunity to make the peer review history publicly available. The record will include editor decision letters (with reviews) and your responses to reviewer comments. If eligible, we will contact you to opt in or out.

We look forward to receiving your revised manuscript.

Kind regards,

Tim Mathes

Academic Editor

PLOS ONE

Journal Requirements:

When submitting your revision, we need you to address these additional requirements.

Please ensure that your manuscript meets PLOS ONE's style requirements, including those for file naming. The PLOS ONE style templates can be found at

https://journals.plos.org/plosone/s/file?id=wjVg/PLOSOne_formatting_sample_main_body.pdf and

https://journals.plos.org/plosone/s/file?id=ba62/PLOSOne_formatting_sample_title_authors_affiliations.pdf

1. Please amend either the title on the online submission form (via Edit Submission) or the title in the manuscript so that they are identical.

[Note: HTML markup is below. Please do not edit.]

Reviewers' comments:

Reviewer's Responses to Questions

**Comments to the Author**

1. Is the manuscript technically sound, and do the data support the conclusions?

The manuscript must describe a technically sound piece of scientific research with data that supports the conclusions. Experiments must have been conducted rigorously, with appropriate controls, replication, and sample sizes. The conclusions must be drawn appropriately based on the data presented. 

Reviewer #1: No

Reviewer #2: Yes

**********

2. Has the statistical analysis been performed appropriately and rigorously? 

Reviewer #1: No

Reviewer #2: Yes

**********

3. Have the authors made all data underlying the findings in their manuscript fully available?

The PLOS Data policy requires authors to make all data underlying the findings described in their manuscript fully available without restriction, with rare exception (please refer to the Data Availability Statement in the manuscript PDF file). The data should be provided as part of the manuscript or its supporting information, or deposited to a public repository. For example, in addition to summary statistics, the data points behind means, medians and variance measures should be available. If there are restrictions on publicly sharing data—e.g. participant privacy or use of data from a third party—those must be specified.

Reviewer #1: No

Reviewer #2: Yes

**********

4. Is the manuscript presented in an intelligible fashion and written in standard English?

PLOS ONE does not copyedit accepted manuscripts, so the language in submitted articles must be clear, correct, and unambiguous. Any typographical or grammatical errors should be corrected at revision, so please note any specific errors here.

Reviewer #1: Yes

Reviewer #2: Yes

**********

5. Review Comments to the Author

Please use the space provided to explain your answers to the questions above. You may also include additional comments for the author, including concerns about dual publication, research ethics, or publication ethics. (Please upload your review as an attachment if it exceeds 20,000 characters)

Reviewer #1: This study seeks to answer an important research question, whether eHealth interventions can improve medication adherence post organ transplant. Given that non-adherence to the immunosuppressant regimens is one of the most common reasons for graft failure, identifying effective interventions to improve non-adherence is paramount to long term graft survival. eHealth interventions provide a potential inexpensive and interactive way to improve adherence, so testing their efficacy is a timely objective. In this systematic review and meta-analysis, the authors conclude that eHealth interventions have similar effects to conventional care, and are a convenient way to improve medication adherence. The methods and assessment of risk of bias appear sound, however there are significant concerns in the analysis, mainly around 4 issues – 1) The assessment of medication adherence; 2) The definitions of outcomes; 3) The appropriateness of the use of meta-analysis; 4) narrow search terms – some new/key studies missing. These issues lead to the conclusion that the authors have applied meta-analysis in the wrong context here, and that narrative synthesis may be a more suitable method for analysing the data from the 5 included studies. Based on this, my recommendation is that major revisions should be applied to answer the specified research question in the most appropriate manner. Please see below for further points to consider when revising the paper.

Abstract:

LINE 45: High risk of bias >50% - it is unusual to categorise Risk of Bias in a %, perhaps it may be more useful to highlight which areas within the ROB assessment showed high ROB?

LINE 50: The Results section of the abstract reports no difference between intervention and control – is it misleading to then say in Conclusions that eHealth interventions are shown to be viable, convenient and portable and easily accessible? Furthermore, this is a deviation from the specified research question, namely to assess the effect of an eHealth intervention – it isn’t mentioned that the aim was to assess the viability, accessibility, portability or convenience, so perhaps re-word this to better reflect the primary research question.

Introduction

Lines 81-83: References #9,10,11 give good figures for rates of non-adherence. Work by Dew et al (Dew MA, et al. Rates and Risk factors for nonadherence to the medical regimen after adult solid organ transplantation. Transplantation 2007;83(7):858-73. [MEDLINE: 17460556]) and Denhaerynck (Denhaerynck K, et al. Prevalence, consequences, and determinants of nonadherence in adult renal transplant patients: a literature review. Transplant International 2005;18(10):1211-33. [MEDLINE: 16162098]) also provide good estimates from pooled data across all organ types, and may be useful references.

Lines 90-91: A definition of what constitutes an eHealth intervention for the purposes of this study is needed here. In general, there is significant variability in the content/type of eHealth interventions used internationally. Not all apps have feedback and counselling in real time, and some are nothing more than simple reminders to take medication. In the 5 studies included in this review, one is a web-based educational intervention (Harrison 2017), and web delivered interventions aren’t listed here in the Introduction as types of eHealth.

Line 97-98: This sentence is unclear, do the authors mean to say “analyse the effects of medication adherence interventions?”

Line 106-107: It’s unclear what the authors mean by “present the direction of development of eHealth intervention for organ transplant recipients”, and how this is related to the meta-analysis? Clarity/rewording of this sentence would be useful.

Methods

Line 114: The last search was August 2019 – this is a rapidly evolving area and some key interventions were published in 2019/2020, perhaps it may be useful to update the search? There are also some studies pre-2019 that may fit the study inclusion criteria, and involve similar eHealth interventions to that which are included in the final analysis below. See below for examples of studies (pre/post 2019) that may fit:

• Hardstaff R, Green K, Talbot D. Measurement of compliance posttransplantation--the results of a 12-month study using electronic monitoring. Transplantation Proceedings 2003;35(2):796-7. [MEDLINE: 12644142] � Similar intervention to Reese 2017

• Henriksson J, Tyden G, Hoijer J, Wadstrom J. A prospective randomized trial on the effect of using an electronic monitoring drug dispensing device to improve adherence and compliance. Transplantation 2016;100(1):203-9. [MEDLINE: 26588006] � Similar intervention to Reese 2017, a web-based platform was used to provide feedback

• Korus M, Cruchley E, Calic M, Gold A, Anthony SJ, Parekh RS, et al. Assessing the acceptability and efficacy of teens taking charge: Transplant-A pilot randomized control trial. Pediatric Transplantation 2020;24(1):e13612. [MEDLINE: 31743564] � is listed as a pilot study, but has similar sample size to studies included in the review

• McGillicuddy JW, Gregoski MJ, Weiland AK, Rock RA, Brunner-Jackson BM, Patel SK, et al. Mobile health medication adherence and blood pressure control in renal transplant recipients: a proof-of-concept randomized controlled trial. JMIR Research Protocols 2013;2(2):e32. [MEDLINE: 24004517] � is listed as a pilot study, but has similar sample size to studies included in the review

• Han A, Min SI, Ahn S, Min SK, Hong HJ, Han N, et al. Mobile medication manager application to improve adherence with immunosuppressive therapy in renal transplant recipients: a randomized controlled trial. PLoS ONE [Electronic Resource] 2019;14(11):e0224595. [MEDLINE: 31689320]

• Kaier K, Hils S, Fetzer S, Hehn P, Schmid A, Hauschke D, et al. Results of a randomized controlled trial analyzing telemedically supported case management in the first year after living donor kidney transplantation - a budget impact analysis from the healthcare perspective. Health Economics Review 2017;7(1):1. [MEDLINE: 28092012]

• Levine D, Torabi J, Choinski K, Rocca JP, Graham JA. Transplant surgery enters a new era: Increasing immunosuppressive medication adherence through mobile apps and smart watches. American journal of surgery 2019;218(1):18-20. [MEDLINE: 30799019]

Line 121: Medication knowledge outcomes are only mentioned here as an outcome, and not described in the Introduction. If this is to be a key focus of the paper, it would be useful to provide the rationale and current evidence for focusing on medication knowledge earlier in the paper.

Line 141-143: When assessing medication adherence in chronic diseases in general, there is poor correlation between self-reported adherence and objectively measured adherence (for example: Moran K, et al. The INCATM (Inhaler Compliance Aid): A comparison with established measures of adherence. Psychology and Health. 2017;32:1266-1287 doi: 10.1080/08870446.2017.1290243.). In the literature regarding medication adherence post-transplant, self-reported medication adherence is poorly associated with clinical outcomes (for example, Scheel J, et al. BMC Nephrology. 2017:18;107 DOI: https://doi.org/10.1186/s12882-017-0517-6), and it is argued that the most accurate way to measure adherence is to include both objective adherence (via clinical measures, such as tacrolimus serum concentration levels, pill counts, prescription refill data) and subjective measures (e.g. self-report via instruments), as this then accounts for both intentional and non-intentional adherence. It seems counterintuitive then to focus only on self-reported adherence via instruments, as stated here. A stronger rationale is required for only including self-reported instruments of medication adherence in the review.

Further to this, the authors state on Line 143 that only studies using self-report (instruments) will be examined, however the meta-analysis presented in the Results section includes 2 studies that have measured adherence through objective means only (Sengpiel, Suhling).

Line 143: The HHS, BAASIS and MACS are mentioned as examples of self-reported adherence instruments. Were studies excluded if they used other self-report measures, for example the MMAS? Were any specific scales for medication knowledge identified prior to the study?

A comment on these measures – the HHS, which is used in the RCT by DeVito Dabbs, is a measure that includes more than just medication adherence. It measures a range of self-monitoring behaviours, not just medication adherence, so including this as a total score in meta-analysis as a measure representing medication adherence isn’t accurate.

Line 149: Why were pilot studies excluded? A stronger rationale for excluding pilot studies is required, particularly as there as good quality trials in this area that are classed as pilot studies, and have similar sample sizes to the 5 included studies.

Line 182: Would it be useful to include sub-group analysis for types of eHealth intervention, given the variability across eHealth interventions?

Line 184: “And total and events”. It would be useful if the authors could clarify what is meant by total and events? In addition to this, a definition of what constitutes non-adherence in each instrument would be useful, as there is significant variation across measures in defining non-adherence.

Results:

Included studies:

• Harrison et al. is a web-based intervention – this isn’t mentioned in the author’s discussion of eHealth earlier in Introduction, therefore a definition of eHealth is necessary as a wide range of interventions are included. For Harrison, the MAC is used - this measure includes a combination of both objective and subjective scores to give an overall adherence score, therefore can authors accurately extract the self-report data from the MAC?

• Sengpiel et al. I’m unclear why this study was included, as adherence is not measured by an instrument, and therefore violates the study inclusion criteria outlined in Methods?

• Suhling et al. – is this an educational intervention delivered via electronic means, or an eHealth intervention? Again, adherence is only measured by an objective outcome, so I’m unclear why this study was included.

Line 244/245: “Medication adherence was divided into two types depending on the cut point”. Was this a cut point per instrument, or an overall cut point? See above re. the need for a definition of non-adherence.

Table 2 -Part A: it is unclear what data is being presented here? The effect size from the meta-analysis is presented in the top row under dichotomous data, but includes both dichotomous and continuous data, from both objective and subjective measures of medication adherence.

Table 2 - Part B: It isn’t clear what these data refer to.

Line 276 onwards: I have concerns about the use of meta-analysis in this instance. The 5 included studies are all combined for a pooled estimate, despite significant heterogeneity in terms of type of measurement (objective adherence and subjective adherence) and heterogeneity of measurement (dichotomous versus continuous data). The pooled estimate is subsequently flawed, and doesn’t accurately answer the research question.

Even amongst the three studies that use self-reported measures, there is heterogeneity between measures, so I’m unsure how confident authors can be in the reliability or accuracy of the outcome of the meta-analysis. For example, Harrison et al. present both self-report and objective data as part of the MACS measure, so how was this entered?

In the forest plot, studies are pooled that are all measuring very different things. The rationale behind pooling all five data sets would need clear explanation.

Discussion

General comment: the conclusions may be overreaching based on the above concerns regarding the accurateness of the presented meta-analysis results.

Line 408 – It is mentioned here that ROB assessment was completed for seven studies, however only 5 studies are included in analysis. Perhaps this is a typo for correction.

Appendices:

Flowchart: Bottom box reports 5 studies included in qualitative synthesis – should this say five studies included in meta-analysis?

Forest plot: See above for concerns regarding suitability of meta-analysis in this instance.

Reviewer #2: EHealth is a timely and important topic to be evaluating currently, and for such serious consequences like adherence in organ transplant patients. I believe this is important research to be reported as societies around the world have been forced to quickly adapt to eHealth technologies currently. However, the meta-analysis has some limitations and a few points I wish were more discussed in the manuscript:

-The introduction flow hits a confusing stride in first stating the negatives of immunosuppressant medications, stating they are a major cause of complications and costs to the healthcare system from their side effects, and then reversing that they are life-saving and must be adhered to. While these points are both true, consider reorganizing this to get to the objective of the study and to validate the importance of adherence to these medications.

-An important consideration that I think was overlooked in the discussion was the fact that you were evaluating eHealth inventions to both what would be considered standard care or to another intervention not involving eHealth. Defining what is standard care versus an actual intervention is a very blurry line that all researchers struggle with and this manuscript should not be rejected due to this. I understand this limitation is due to the availability of literature and how these studies are reported, but it must be discussed more. If you look at the effect sizes based on the individual studies, the two eHealth interventions that showed higher effectiveness were versus what is basically a one-time consultation that would happen in standard care. The other eHealth interventions that were not as effective were compared to what seems to be more advanced interventions involving multiple pharmacist meetings or having a wireless pill container. I think this is really important to note that while these "standard" interventions happening face-to-face may be just as effective as eHealth, their consumption of resources and often times inaccessibility are a reason for advocating for eHealth interventions.

-The I squared of 0 is very biased due to their only being five studies included. This is an important limitation and consideration in the heterogeneity being discussed. I do appreciate the detail on the measures of adherence used in all studies. If I understand correctly, three used a questionnaire and two used drug concentration? While both valid measures of adherence, they would differ considerably.

**********

6. PLOS authors have the option to publish the peer review history of their article (what does this mean?). If published, this will include your full peer review and any attached files.

If you choose “no”, your identity will remain anonymous but your review may still be made public.

**Do you want your identity to be public for this peer review?** For information about this choice, including consent withdrawal, please see our Privacy Policy.

Reviewer #1: No

Reviewer #2: Yes: Elyssa K Wiecek

[NOTE: If reviewer comments were submitted as an attachment file, they will be attached to this email and accessible via the submission site. Please log into your account, locate the manuscript record, and check for the action link "View Attachments". If this link does not appear, there are no attachment files to be viewed.]

While revising your submission, please upload your figure files to the Preflight Analysis and Conversion Engine (PACE) digital diagnostic tool, https://pacev2.apexcovantage.com/. PACE helps ensure that figures meet PLOS requirements. To use PACE, you must first register as a user. Registration is free. Then, login and navigate to the UPLOAD tab, where you will find detailed instructions on how to use the tool. If you encounter any issues or have any questions when using PACE, please email us at figures@plos.org. Please note that Supporting Information files do not need this step.

Attachment Submitted filename: L.Mellon PLoS One review.docx Click here for additional data file.

# Author comment: Author response to Decision Letter 0

**DOI:** 10.1371/journal.pone.0241857.r002

27 May 2020

We did our best to revise as you requested. I am grateful that your comments have improved the quality of our paper. Thank you very much.

Attachment Submitted filename: Response to reviewers.docx Click here for additional data file.

# Peer review: Decision Letter 1

**DOI:** 10.1371/journal.pone.0241857.r003

**Authors:** Tim Mathes

16 Jul 2020

PONE-D-20-07165R1

Effectiveness of eHealth interventions for improving medication adherence of organ transplant patients: A systematic review and meta-analysis

PLOS ONE

Dear Dr. Seo,

Thank you for submitting your manuscript to PLOS ONE. After careful consideration, we feel that it has merit but does not fully meet PLOS ONE’s publication criteria as it currently stands. Therefore, we invite you to submit a revised version of the manuscript that addresses the points raised during the review process.

Some meta-analysis were performed, despite statistical significant test for heterogeneity (meta-analysis A and D). In its current form the analysis adequate. The authors should either only perform a structured narrative synthesis or perform an in depth analysis of  heterogeneity in these cases.

In addition, please structure the manuscript as suggested in PRISMA (e.g. the section on risk of bias before the results).

Please submit your revised manuscript by Aug 30 2020 11:59PM. If you will need more time than this to complete your revisions, please reply to this message or contact the journal office at plosone@plos.org. When you're ready to submit your revision, log on to https://www.editorialmanager.com/pone/ and select the 'Submissions Needing Revision' folder to locate your manuscript file.

Please include the following items when submitting your revised manuscript:

- A rebuttal letter that responds to each point raised by the academic editor and reviewer(s). You should upload this letter as a separate file labeled 'Response to Reviewers'.
- A marked-up copy of your manuscript that highlights changes made to the original version. You should upload this as a separate file labeled 'Revised Manuscript with Track Changes'.
- An unmarked version of your revised paper without tracked changes. You should upload this as a separate file labeled 'Manuscript'.

If you would like to make changes to your financial disclosure, please include your updated statement in your cover letter. Guidelines for resubmitting your figure files are available below the reviewer comments at the end of this letter.

If applicable, we recommend that you deposit your laboratory protocols in protocols.io to enhance the reproducibility of your results. Protocols.io assigns your protocol its own identifier (DOI) so that it can be cited independently in the future. For instructions see: http://journals.plos.org/plosone/s/submission-guidelines#loc-laboratory-protocols

We look forward to receiving your revised manuscript.

Kind regards,

Tim Mathes

Academic Editor

PLOS ONE

[Note: HTML markup is below. Please do not edit.]

Reviewers' comments:

Reviewer's Responses to Questions

**Comments to the Author**

1. If the authors have adequately addressed your comments raised in a previous round of review and you feel that this manuscript is now acceptable for publication, you may indicate that here to bypass the “Comments to the Author” section, enter your conflict of interest statement in the “Confidential to Editor” section, and submit your "Accept" recommendation.

Reviewer #1: (No Response)

Reviewer #2: All comments have been addressed

**********

2. Is the manuscript technically sound, and do the data support the conclusions?

The manuscript must describe a technically sound piece of scientific research with data that supports the conclusions. Experiments must have been conducted rigorously, with appropriate controls, replication, and sample sizes. The conclusions must be drawn appropriately based on the data presented. 

Reviewer #1: Partly

Reviewer #2: Yes

**********

3. Has the statistical analysis been performed appropriately and rigorously? 

Reviewer #1: No

Reviewer #2: Yes

**********

4. Have the authors made all data underlying the findings in their manuscript fully available?

The PLOS Data policy requires authors to make all data underlying the findings described in their manuscript fully available without restriction, with rare exception (please refer to the Data Availability Statement in the manuscript PDF file). The data should be provided as part of the manuscript or its supporting information, or deposited to a public repository. For example, in addition to summary statistics, the data points behind means, medians and variance measures should be available. If there are restrictions on publicly sharing data—e.g. participant privacy or use of data from a third party—those must be specified.

Reviewer #1: Yes

Reviewer #2: Yes

**********

5. Is the manuscript presented in an intelligible fashion and written in standard English?

PLOS ONE does not copyedit accepted manuscripts, so the language in submitted articles must be clear, correct, and unambiguous. Any typographical or grammatical errors should be corrected at revision, so please note any specific errors here.

Reviewer #1: Yes

Reviewer #2: Yes

**********

6. Review Comments to the Author

Please use the space provided to explain your answers to the questions above. You may also include additional comments for the author, including concerns about dual publication, research ethics, or publication ethics. (Please upload your review as an attachment if it exceeds 20,000 characters)

Reviewer #1: The authors have made significant amendments to the paper that have strengthened it considerably. I still disagree however with the use of meta-analysis, given the significant heterogeneity across the measures used here. The authors have given their rationale for proceeding with meta-analysis, and therefore I have flagged some concerns where the authors need to provide more information to flag the significant heterogeneity between studies and their method of measurement.

Page 16: Outcomes section: I think it would be useful to highlight in this section the significant heterogeneity across the studies in how adherence was measured. The authors have provided more information here on how non-adherence was categorised, but it is still vague in parts. For example, how were the tacrolimus blood concentrations levels categorised as non-adherent across studies? Different authors use different algorithms, so it is important to detail the variance in categorisation to readers. Similarly, for the self-reported questionnaires, more detail is required here to explain to readers the heterogeneity across measures.

Table 2: I’m unsure as to what is being presented here for medication knowledge. Given that there was one study with a dichotomous measure of knowledge, and one with a continuous measure, I’m presuming this wasn’t meta-analysed, but as it is in a table with data from meta-analysis for medication adherence, this is misleading. I suggest reporting findings for knowledge in text alone for clarity.

Page 26: The limitations of including the HHS as an overall medication adherence score are addressed, however it isn’t acknowledged that the MACS is also a multidimensional adherence scale and including this as a total score may produce misleading results.

Line 457: It may be useful to comment that alongside high quality studies, there is a need for standardised measurement and definitions of adherence to improve the quality of the research in this area.

Figure 2: Figs B & D – I fail to see the usefulness of the statistics presented here in these two estimates of effect size. Fig D, a pooled estimate from 2 studies, with an I2 of 97% is like comparing apples with oranges! It would be more useful to the reader to report this in narrative synthesis. Perhaps Chapter 10 of the Cochrane handbook may provide guidance on the appropriateness of the data for meta-analysis.

For figs A-D, it would be useful to add a note to each study in RevMan listing the method of adherence measurement (e.g. name of questionnaire). A title for each is also required – it’s not clear from current figure what C&D represent?

Reviewer #2: Well done to the authors on the corrections. I believe the separation of the meta-analysis was a wise decision and made the results more technically sound. I am also much happier with the way the interventions and comparisons were described as well as measures of adherence.

**********

7. PLOS authors have the option to publish the peer review history of their article (what does this mean?). If published, this will include your full peer review and any attached files.

If you choose “no”, your identity will remain anonymous but your review may still be made public.

**Do you want your identity to be public for this peer review?** For information about this choice, including consent withdrawal, please see our Privacy Policy.

Reviewer #1: **Yes: **Lisa Mellon

Reviewer #2: **Yes: **Elyssa K Wiecek

[NOTE: If reviewer comments were submitted as an attachment file, they will be attached to this email and accessible via the submission site. Please log into your account, locate the manuscript record, and check for the action link "View Attachments". If this link does not appear, there are no attachment files.]

While revising your submission, please upload your figure files to the Preflight Analysis and Conversion Engine (PACE) digital diagnostic tool, https://pacev2.apexcovantage.com/. PACE helps ensure that figures meet PLOS requirements. To use PACE, you must first register as a user. Registration is free. Then, login and navigate to the UPLOAD tab, where you will find detailed instructions on how to use the tool. If you encounter any issues or have any questions when using PACE, please email PLOS at figures@plos.org. Please note that Supporting Information files do not need this step.

# Author comment: Author response to Decision Letter 1

**DOI:** 10.1371/journal.pone.0241857.r004

21 Aug 2020

Thank you for your valuable comments regarding our manuscript. We carried out a point-by-point revision of the manuscript following the academic editor and reviewers’ comments, and indicated the edited sentences in red font. We have made an earnest effort to revise the manuscript in accordance with the academic editor and reviewers’ suggestions.

Attachment Submitted filename: Response to reviewers.docx Click here for additional data file.

# Peer review: Decision Letter 2

**DOI:** 10.1371/journal.pone.0241857.r005

**Authors:** Tim Mathes

22 Oct 2020

Effectiveness of eHealth interventions for improving medication adherence of organ transplant patients: A systematic review and meta-analysis

PONE-D-20-07165R2

Dear Dr. Seo,

We’re pleased to inform you that your manuscript has been judged scientifically suitable for publication and will be formally accepted for publication once it meets all outstanding technical requirements.

Within one week, you’ll receive an e-mail detailing the required amendments. When these have been addressed, you’ll receive a formal acceptance letter and your manuscript will be scheduled for publication.

An invoice for payment will follow shortly after the formal acceptance. To ensure an efficient process, please log into Editorial Manager at http://www.editorialmanager.com/pone/, click the 'Update My Information' link at the top of the page, and double check that your user information is up-to-date. If you have any billing related questions, please contact our Author Billing department directly at authorbilling@plos.org.

If your institution or institutions have a press office, please notify them about your upcoming paper to help maximize its impact. If they’ll be preparing press materials, please inform our press team as soon as possible -- no later than 48 hours after receiving the formal acceptance. Your manuscript will remain under strict press embargo until 2 pm Eastern Time on the date of publication. For more information, please contact onepress@plos.org.

Kind regards,

Tim Mathes

Academic Editor

PLOS ONE

Additional Editor Comments (optional):

Reviewers' comments:

Reviewer's Responses to Questions

**Comments to the Author**

1. If the authors have adequately addressed your comments raised in a previous round of review and you feel that this manuscript is now acceptable for publication, you may indicate that here to bypass the “Comments to the Author” section, enter your conflict of interest statement in the “Confidential to Editor” section, and submit your "Accept" recommendation.

Reviewer #1: All comments have been addressed

**********

2. Is the manuscript technically sound, and do the data support the conclusions?

The manuscript must describe a technically sound piece of scientific research with data that supports the conclusions. Experiments must have been conducted rigorously, with appropriate controls, replication, and sample sizes. The conclusions must be drawn appropriately based on the data presented. 

Reviewer #1: Yes

**********

3. Has the statistical analysis been performed appropriately and rigorously? 

Reviewer #1: Yes

**********

4. Have the authors made all data underlying the findings in their manuscript fully available?

The PLOS Data policy requires authors to make all data underlying the findings described in their manuscript fully available without restriction, with rare exception (please refer to the Data Availability Statement in the manuscript PDF file). The data should be provided as part of the manuscript or its supporting information, or deposited to a public repository. For example, in addition to summary statistics, the data points behind means, medians and variance measures should be available. If there are restrictions on publicly sharing data—e.g. participant privacy or use of data from a third party—those must be specified.

Reviewer #1: Yes

**********

5. Is the manuscript presented in an intelligible fashion and written in standard English?

PLOS ONE does not copyedit accepted manuscripts, so the language in submitted articles must be clear, correct, and unambiguous. Any typographical or grammatical errors should be corrected at revision, so please note any specific errors here.

Reviewer #1: Yes

**********

6. Review Comments to the Author

Please use the space provided to explain your answers to the questions above. You may also include additional comments for the author, including concerns about dual publication, research ethics, or publication ethics. (Please upload your review as an attachment if it exceeds 20,000 characters)

Reviewer #1: This is an important review in the area of medication adherence post-transplant. The authors have done an excellent job of revising the results of the study, and I am satisfied that the methods of analysis now match the quality and heterogeneity of data in the review.

**********

7. PLOS authors have the option to publish the peer review history of their article (what does this mean?). If published, this will include your full peer review and any attached files.

If you choose “no”, your identity will remain anonymous but your review may still be made public.

**Do you want your identity to be public for this peer review?** For information about this choice, including consent withdrawal, please see our Privacy Policy.

Reviewer #1: **Yes: **Lisa Mellon

# Editor report: Acceptance letter

**DOI:** 10.1371/journal.pone.0241857.r006

**Authors:** Tim Mathes

26 Oct 2020

PONE-D-20-07165R2 

Effectiveness of eHealth interventions for improving medication adherence of organ transplant patients: A systematic review and meta-analysis 

Dear Dr. Seo:

I'm pleased to inform you that your manuscript has been deemed suitable for publication in PLOS ONE. Congratulations! Your manuscript is now with our production department. 

If your institution or institutions have a press office, please let them know about your upcoming paper now to help maximize its impact. If they'll be preparing press materials, please inform our press team within the next 48 hours. Your manuscript will remain under strict press embargo until 2 pm Eastern Time on the date of publication. For more information please contact onepress@plos.org.

If we can help with anything else, please email us at plosone@plos.org. 

Thank you for submitting your work to PLOS ONE and supporting open access. 

Kind regards, 

PLOS ONE Editorial Office Staff

on behalf of

Dr. Tim Mathes 

Academic Editor

PLOS ONE
